# Macrophage plasticity and metabolic control in muscle repair and disease

**DOI:** 10.3389/fimmu.2026.1872583

**Published:** 2026-08-25

**Authors:** Sarah Tiufekchiev-Grieco, James S. Novak, Jyoti K. Jaiswal

**Affiliations:** 1 Center for Precision Medicine and Genomics Research, Children’s National Research Institute, Children’s National Research and Innovation Campus, Children’s National Hospital, Washington, DC, United States; 2 Integrated Biomedical Sciences, The George Washington University School of Medicine and Health Sciences, Washington, DC, United States; 3 Department of Pediatrics, The George Washington University School of Medicine and Health Sciences, Washington, DC, United States; 4 Department of Biochemistry and Molecular Medicine, The George Washington University School of Medicine and Health Sciences, Washington, DC, United States

**Keywords:** macrophage, metabolism, mitochondria, muscle, myogenesis, myopathy

## Abstract

Inflammation is a tightly regulated process essential for skeletal muscle repair, and its dysregulation contributes to chronic disease and impaired regeneration. Following injury, muscle repair involves a coordinated immune response initiated by neutrophil infiltration, followed by macrophage recruitment and diversification. Rather than existing as discrete subsets, macrophages span a continuum of functional states that evolve over time in response to local environmental cues, enabling transitions from clearing debris and pro-inflammatory signaling to supporting resolution of inflammation, and remodeling and regeneration of the tissue. This functional plasticity is closely linked to intracellular metabolic programs. In this review, we examine how metabolic pathways, particularly the balance between glycolysis and oxidative phosphorylation, govern macrophage behavior through epigenetic mechanisms, thereby coupling cellular metabolism to inflammatory and regenerative gene expression. We further explore how these interconnected pathways are disrupted in chronic inflammatory muscle diseases, including muscular dystrophies. Recent transcriptomic studies highlight pathogenic macrophage populations with altered metabolic and epigenetic profiles that contribute to fibrosis and impaired regeneration. By integrating findings from both acute injury and chronic disease contexts, we provide a framework to explore macrophage function through a metabolic and epigenetic lens and discuss emerging strategies aimed at restoring macrophage plasticity and promoting the resolution of inflammation in muscle disease.

## Introduction

Inflammation is a unique protective response in multicellular organisms that operates at a tissue, cellular, and molecular level to defend against injury, infection, and cellular damage (1, 2). While often associated with pain or illness, inflammation is essential for tissue healing and recovery, only becoming detrimental when it is uncontrolled, and becomes excessive or persistent, as is the case in a range of chronic health conditions (3–5). While inflammatory response is minimally detected in resting skeletal muscles, inflammation plays a critical and highly regulated role during recovery from strenuous activity and injury (6–8). A variety of immune cells, including leucocytes (e.g. neutrophils and macrophages) and lymphocytes work in concert to receive and transmit signals upon injury that regulate their temporal dynamics and regeneration of the injured muscle (9, 10). This acute inflammatory response helps remove damaged tissue and activate tissue regenerative processes to recover the lost muscle fibers (10–12). When properly controlled, inflammation allows muscle adaptation and improved function, while chronic inflammation, as seen in the context of disease, can interfere with recovery and contribute to muscle weakness and loss over time (10).

In the context of muscle repair, macrophages are widely studied for their role in clearing debris and supporting regeneration. Following their recruitment and activation, macrophages transition through a gradient of phenotypes ranging from pro-inflammatory to pro-regenerative states, which is guided by their niche, metabolism and epigenetic alterations (13–16). The binary pro-inflammatory (M1) and pro-regenerative (M2) macrophage profiles have commonly been used to describe the major polarization of macrophages throughout the waves of inflammation after injury (14). This M1 and M2 nomenclature fails to account for the complexity and diversity of macrophage functional profiles following acute injury (17). Thus, to better characterize this diversity, we propose the term *Acute Inflammatory Macrophages (AIMs)* to describe the range of transitory states of macrophages during the initial, pro-inflammatory phase of inflammation. Additionally, as the function of macrophages shifts to support tissue repair, we introduce use of the term *Acute Regenerative Macrophages (ARMs)* to describe these latter transitory phenotypes. Distinct from these acute (transitory) functional profiles, macrophages also appear in a range of chronically inflamed states due to systemic (e.g. metabolic imbalance, aging, etc.) or tissue-specific (e.g. myopathy, muscular dystrophy, etc.) factors. While several of their features overlap with AIMs, including the expression of certain transcripts, their stable persistence and accumulation within chronically-injured tissues, uniquely distinguish them from AIMs, and thus we suggest use of the label *Terminal Inflammatory Macrophages (TIMs)* to describe this pathogenic fate.

**Figure 1:**
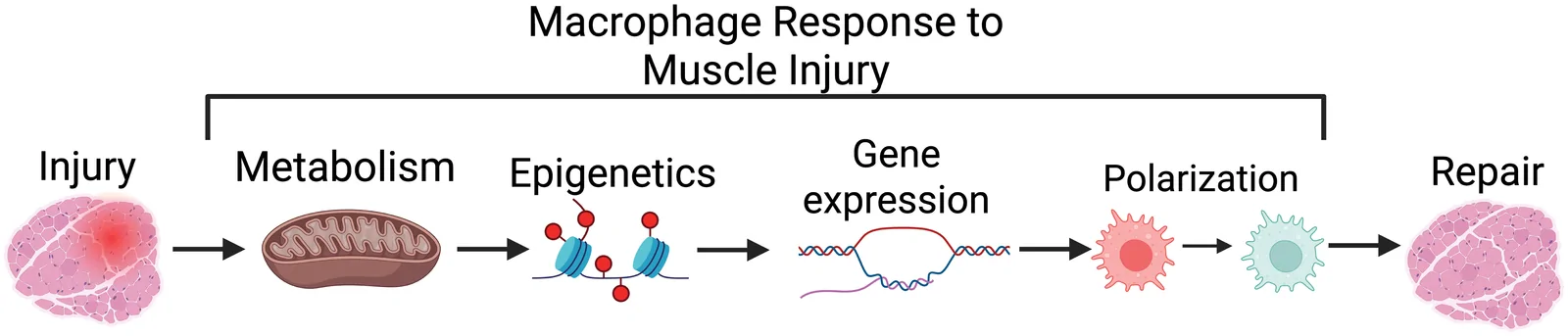
Macrophage polarization is impacted by metabolic and epigenetic factors. Following injury, the muscle niche is altered and can impact the metabolism and therefore downstream epigenetic activity in macrophages. These changes alter gene expression and overall macrophage function, contributing to the interplay of several biological processes that must occur in a balanced and temporal manner to allow for proper inflammatory response, resolution and return to homeostasis.

In this review, we will discuss the continuum of macrophage function in healthy acute muscle repair and describe the metabolic-epigenetic axis which helps regulate macrophage polarization and properties (**Figure 1**). Further, we will discuss the metabolic-epigenetic profiles of macrophages involved in chronic inflammation, revealing therapeutic avenues to support resolution of chronic inflammation and prevent muscle loss.

### Temporal regulation of inflammatory responses during acute injury and repair of skeletal muscle

A healthy muscle niche is comprised of a complex network of muscle and stromal cells including satellite cells (SCs), endothelial cells (ECs), fibroadipogenic progenitor cells (FAPs), and a variety of immune cells, all of which communicate with one another to maintain homeostasis in health or in response to acute injury (18, 19). Given the mechanical and load-bearing nature of muscle, it is unavoidable that there will be acute bouts of injury even in the healthiest context. Acute injury causes a temporary disruption to the homeostatic muscle niche. The way the niche responds to this disruption, particularly the temporal dynamics of inflammatory response and resolution of injury, governs the efficiency of tissue remodeling and regeneration following acute injury, and any deviation in stromal cell responses can delay repair and the return to homeostasis (10, 12, 20).

**Figure 2:**
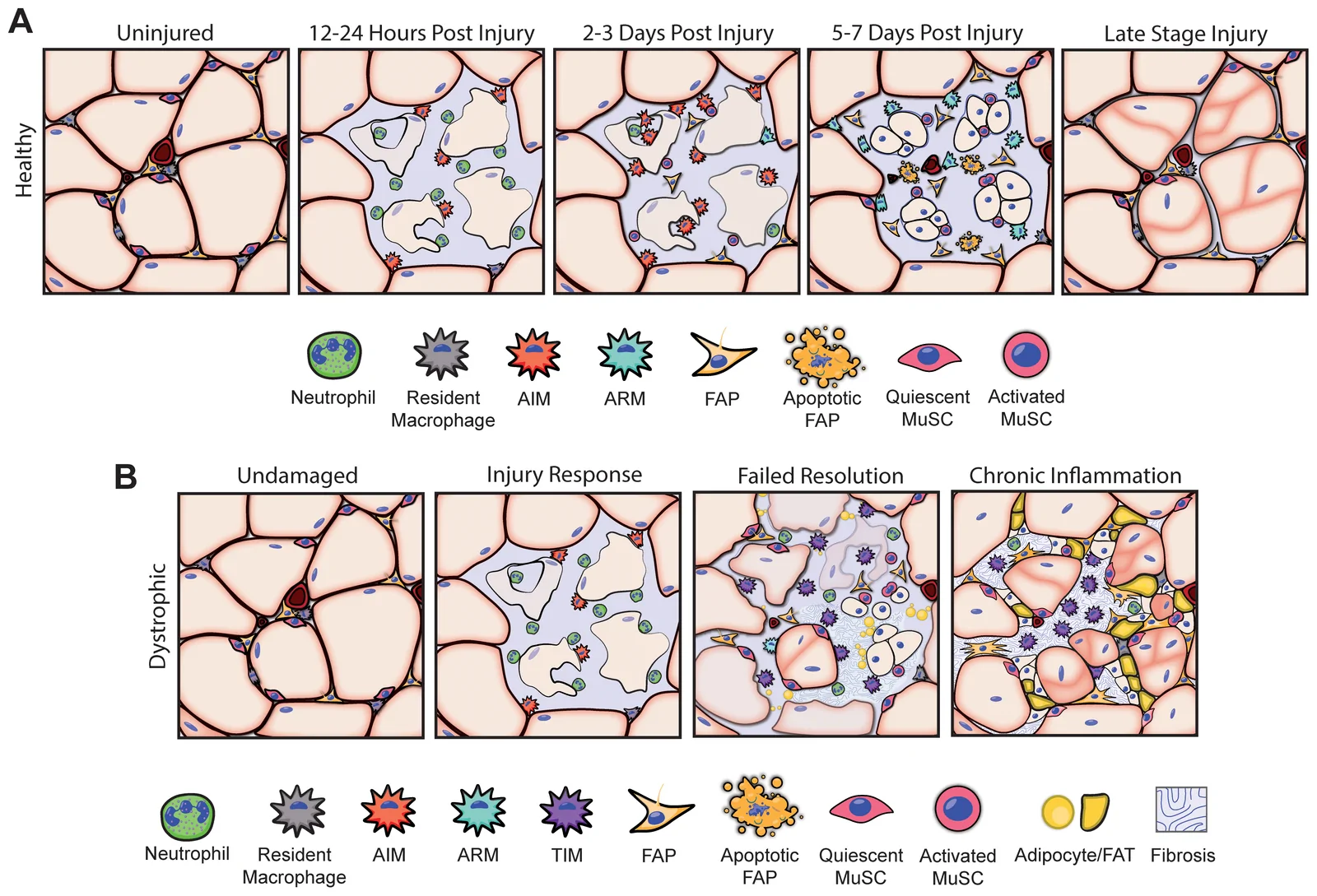
Cellular choreography following acute injury in skeletal muscle. Muscle injury triggers various cells to accumulate transiently for proper inflammatory and regenerative interactions for the eventual repair of the injured muscle. **(A)** In healthy muscle neutrophil accumulation peaks 12-24 hours following injury and they begin recruiting *acute inflammatory macrophages* (AIMs) to the site of injury. By 2-3 days, accumulation of AIMs peaks, clearing damaged myofibers and AIMs begin to adopt a more regenerative phenotype. By 5-7 days post injury, *acute regenerative macrophages* (ARMs) are abundant where they continue to promote the expansion of FAPs and the final stages of myofiber regeneration through SC differentiation and fusion by late timepoints post injury. **(B)** In dystrophic conditions, after injury response is mounted, AIMs fail to resolve toward an ARM like phenotype and *terminal inflammatory macrophages* (TIMs) persist at the site of injury, leading to chronic inflammation and fibrotic and adipogenic accumulation rather than regeneration of muscle. This schematic has been adapted from Hogarth et al. (21).

To understand the multifaceted role of inflammation within the context of acute muscle repair, it is important to recognize the temporal nature of inflammatory signaling through immune cell accumulation and clearance (**Figure 2A**). The first immune cells to enter the damaged muscle are neutrophils, which peak over 12-24 hours after injury (10, 12, 18, 20, 22) (**Figure 2A**). Recruitment of neutrophils from the vasculature after an injury is driven by the sudden and localized release of damage-associated molecular patterns (DAMPs) from the injured myofibers. Tissue-resident macrophages recognize these DAMPs and release inflammatory chemoattractants, including CXCL1/2 and 8, which help recruit neutrophils to the site of injury where they initiate the process of clearing debris (23–25). Additionally, they help reconstruct the damaged vascular network through activation of VEGF signaling to allow local access to required nutrients and an avenue for additional circulating myeloid and lymphoid cells to reach the damaged space (26). These activated neutrophils release factors like sIL-6Rα, which combined with local IL-6, lead to secretion of CCL2, promoting monocyte recruitment and differentiation into macrophages within the tissue for the next stage of the inflammatory process (25, 27–29) (**Figure 2A**). Although neutrophils are known to clear out relatively quickly after their initial recruitment, in some cases of injury or in disease, neutrophils may linger in areas of damage and continue to secrete pro-inflammatory signals, which can impede the temporal dynamics of inflammatory and repair processes mediated by macrophages and other stromal cell types thereafter (25, 30, 31) (**Figure 2B**).

In healthy response to acute injury, after neutrophil activation, circulating monocytes and macrophages infiltrate the damaged area rich with proinflammatory cytokines, like IFNγ and TNF-α (25). These activated monocytes then polarize to take on an *acute inflammatory macrophage* (AIM) profile, characterized by high Ly6C expression and heightened secretion of pro-inflammatory cytokines (32, 33). After the initial wave of recruitment of Ly6C positive/high monocytes and AIM presence, the overall macrophage profile within the injured niche transitions towards a Ly6C negative/low state (33), where these *acute regenerative macrophages* (ARMs) support ECM remodeling and myofiber regeneration (16, 34).

The abundance of inflammatory macrophages (AIMs) peak within 3 days post injury and their phagocytic activities help clear additional debris as neutrophils depart the damaged site (10, 16, 35, 36) (**Figure 2A**). AIMs also release chemokines and cytokines which support subsequent pro-inflammatory processes including phagocytosis, while also activating myogenic processes via SC interactions (10, 11, 37–39). Several factors are involved in facilitating the switch of pro-inflammatory AIMs toward pro-regenerative ARMs, including molecular effectors like MKP1, AMPK, METRNL and IGF-1, in addition to the act of phagocytosing muscle fiber debris, which in itself stimulates AIM polarization towards a pro-regenerative phenotype (14, 40–43). These dynamics indicate the highly plastic nature of the macrophages in various states between AIMs and ARMs.

ARMs, which peak in abundance within the acutely injured muscle niche 5-7 days post injury, support remodeling of the local environment through stromal cell interactions and transient ECM deposition to facilitate myogenesis and the process of regeneration (10, 12, 21, 35, 44, 45) (**Figure 2A**). These macrophages differ from AIMs in their receptor expression, metabolism, as well as cytokine and chemokine production. Timely polarization of AIM- and ARM-like profiles is essential to the acute inflammatory response, as expression of a variety of anti-inflammatory cytokines like *Il4, Il10* and *Tgfb* by ARMs not only reduces pro-inflammatory signaling but also actively halts recruitment of neutrophils and additionally promotes neutrophil apoptosis (35, 46). This is disrupted during chronic muscle inflammation caused by repeated injury and failed resolution of inflammation, resulting in accumulation and persistence of *terminal inflammatory macrophages* (TIMs). These cells share some features with AIMs and ARMs, but their inability to resolve contributes to improper muscle repair and long-term inflammation, driving the pathology of chronic inflammatory diseases like muscular dystrophies (47–50) (**Figure 2B**).

Along with circulating monocytes, which are recruited to the site of damage, prompted to mature into macrophages, and then activated towards inflammatory states, the tissue resident macrophages also participate in regulation of inflammation (51, 52). Resident macrophages are established in embryogenesis and remain in skeletal muscle through adulthood by self-renewal (53–55). Additional resident macrophages can also arise through replenishment via circulating blood monocytes (53, 55). Skeletal muscle resident macrophages help maintain homeostasis by controlling inflammation after small injuries, but their role after major acute injury, when many other macrophages enter the tissue, is not fully understood (51, 56, 57). However, the self-renewing resident macrophages function in the clearance of apoptotic cells in cases of acute injury, and also impact muscle fiber composition in instances of chronic mild injury (54). Additionally, resident macrophages can be activated towards alternative polarization profiles, which indicates that AIMs, ARMs and TIMs do not necessarily arise solely from the circulating monocytes, as demonstrated following inhibition of monocyte infiltration into damaged dystrophic muscle (35, 53, 58). Additional immune cells, including regulatory T lymphocytes and eosinophils also support muscle repair, but the monocytes and macrophages represent the majority of immune cells present in an injured muscle (18, 35, 59).

### Macrophage and stromal cell interactions in damaged skeletal muscle repair

**Figure 3:**
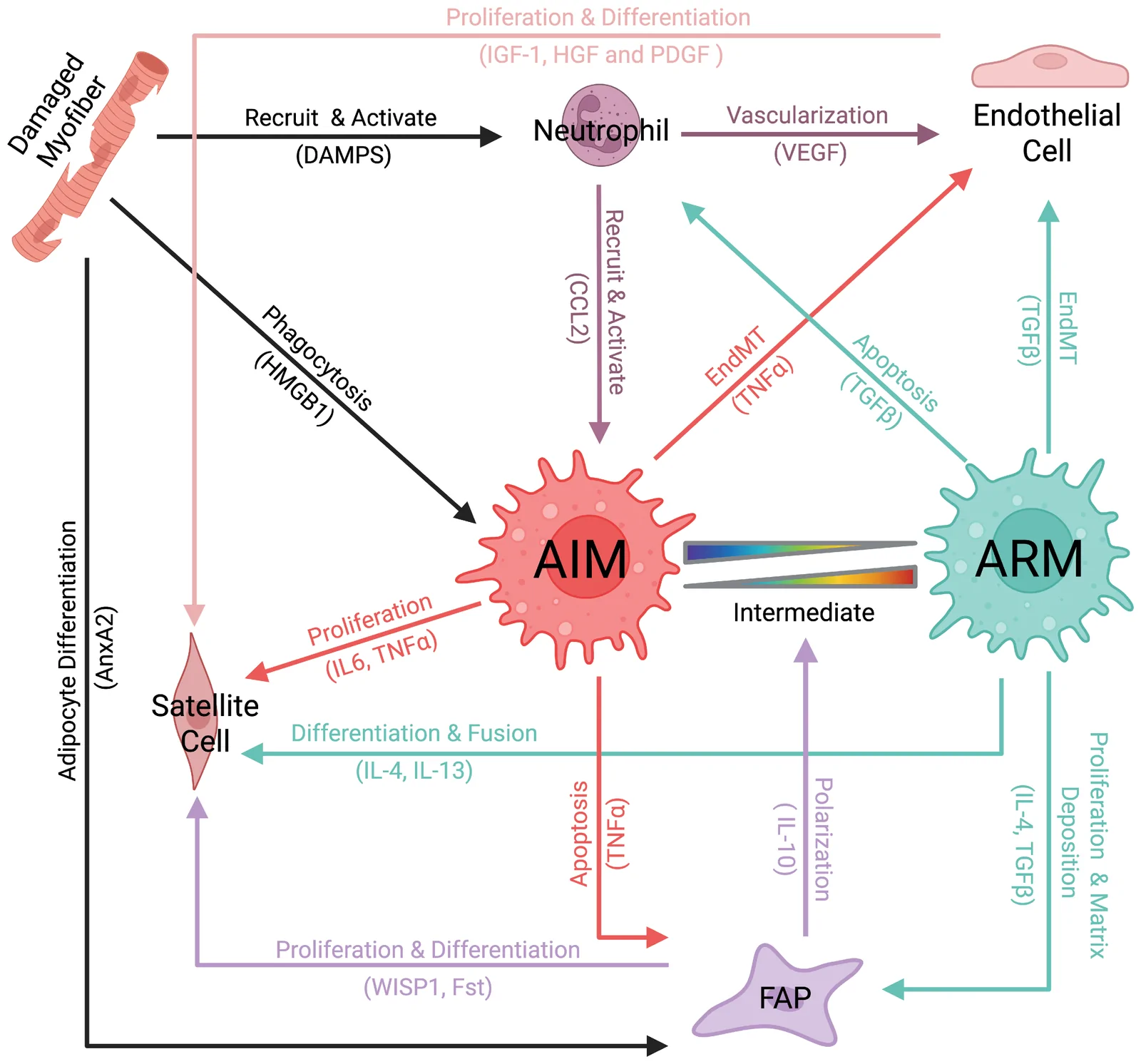
Stromal cell interactions during acute muscle repair. Macrophages and other cells within the injured muscle niche interact and create a complex regulatory network to allow for the proper balance of pro and anti-inflammatory signaling. The schematic highlights some of the pathways involved in signaling interactions between damaged myofibers, neutrophils, AIMs, ARMs, ECs, FAPs, and SCs following an acute injury in the muscle.

In addition to direct functional impacts on inflammatory processes in the injured muscle, over the course of repair, macrophages interact with various other stromal cell types critical for the repair of acutely injured muscle (**Figure 2**). Of the various non-immune, muscle-resident cells that macrophages interact with, critical cell types include SCs, ECs, and FAPs. Macrophages support the proliferation and/or differentiation of these cells within the damaged niche (10, 16, 45, 60, 61). These interactions are critical to the proper regeneration of muscle as well as remodeling of the ECM and vascular network and are regulated by distinct signaling pathways (**Figure 3**).

Macrophage interactions with SCs allow for balanced expansion and differentiation of SCs for the regeneration of damaged muscle fibers. AIMs promote SC proliferation, and inhibit their differentiation through the release of cytokines like IL-6 and TNF-α, while secretion of anti-inflammatory cytokines released by ARMs, including IL-4 and IL-13, stimulate myoblast differentiation and fusion (16, 62–67) (**Figure 3**). In healthy steady state, SCs also recruit ECs via secretion of VEGFA, which in turn aid in the maintenance of SC quiescence (68). (**Figure 3**).

FAPs are muscle-resident mesenchymal stem cells that proliferate upon injury, where they can differentiate into fibroblasts or adipocytes (69–72). Their fate is tightly regulated by interactions with factors secreted in the muscle environment from cells including macrophages and SCs (71, 73, 74). Early after injury, FAPs rapidly proliferate and adopt a pro-regenerative phenotype enriched in follistatin (Fst), and secrete chemokines that influence immune cell fate and support myogenic cells (75) (**Figure 3**). As regeneration progresses, FAPs differentiate into fibroblasts to restore the ECM of the repairing muscle, while the absence of FAPs impairs myogenesis (76). Like macrophages, the change in FAP accumulation and activity during repair is tightly regulated. Inflammation supports FAP abundance and differentiation, such that early on following injury, eosinophils promote FAP proliferation via IL-4/IL-13 signaling, while later AIMs promote apoptotic FAP clearance through production of TNF-α (61, 73) (**Figure 3**). Early in the inflammatory phase, AIMs secrete matrix remodeling enzymes (e.g. MMPs) to locally degrade the ECM, while ARMs support FAP differentiation through TGFβ1 signaling (61) (**Figure 3**). This blocks TNF–induced FAP apoptosis and supports FAPs to differentiate and secrete the matrix around the newly forming myofibers (61). In chronically inflamed tissues, macrophage-FAP crosstalk causes accumulation and fibrotic differentiation of FAPs via TGFβ and IL-4 signaling (61, 75, 77). Depletion of ARMs or inhibition of TGFβ1 relieves myogenic suppression of FAP-derived Fst (75). Additionally macrophages and injured myofibers release Annexin A2 (AnxA2) which can promote differentiation of FAPs into adipocytes (78, 79) (**Figure 3**).

Along with being regulated by macrophages, FAPs regulate various cells in the muscle niche. For instance, FAPs increase their expression of *Il10* following muscle injury which is a cytokine involved in initiating an ARM-like polarization of macrophages (16, 80, 81). Additionally, FAPs influence healthy SC expansion via WISP1, as well as their differentiation through Fst signaling (82, 83), while suppressing myogenic differentiation in chronically inflamed tissues (84). This highlights the tightly coordinated interplay between immune and myogenic responses, which is disrupted in diseased muscles (21, 84, 85) (**Figure 3**). In healthy muscles, blocking monocyte recruitment to injury sites contributes to impaired FAP clearance and dysregulated matrix deposition (e.g., collagen overproduction) and fibrosis, while restoring regulation of this in diseased muscles improves the efficiency of myogenesis (58, 61, 84–86).

Macrophage interactions with ECs impact vascularization after injury – culturing ECs with macrophages enhances formation of blood vessels through secreted factors (35, 87, 88). (**Figure 3**). Priming ECs with AIMs supports angiogenesis (87, 89), but increased ARM recruitment has been associated with improved vascular remodeling and an anti-angiogenic role (87, 90, 91). These findings support macrophage involvement in angiogenic signaling, but this depends on complex local and temporal interactions. Macrophage recruitment and polarization supports EC function and phenotype (35, 92). This includes impact on ECs such as prevention of endothelial to mesenchymal transition (EndMT) causing accumulation of ECs that support fibrosis (92). In particular, macrophage expression of *Tnfα* and *Spp1* contributes to EndMT through TGFβ signaling, which is also activated in muscular dystrophies due to impaired AIM to ARM transition. This impacts EC crosstalk and causes excessive EndMT, fibrotic accumulation, and ineffective muscle regeneration (61, 92–94) (**Figure 3**). In addition to their interaction with macrophages, ECs also contribute to signaling with other stromal cells further illustrating the critical interplay of various cell-cell interactions within the muscle niche. For example, ECs support SC expansion and differentiation through secretion of growth factors like IGF-1, HGF and PDGF (95–97) (**Figure 3**).

Along with their role in signaling to other stromal cell populations in the muscle niche, macrophages interact with and are influenced by myofibers themselves. Necrotic myofibers release signals including HMGB1 which recruit phagocytic cells to induce clearance of damaged myofiber elements (98, 99) (**Figure 3**). AIMs recognize these signals and clear debris, which subsequently induces macrophages to transition from AIM to ARM-like states by decreasing TNF-α secretion and increasing TGFβ secretion, promoting progression towards resolution of inflammation and muscle regeneration (16).

### Influence of the metabolic-epigenetic axis on macrophage polarization during muscle repair

Given the variety of roles macrophages play in the process of healing, their polarization needs to be carefully regulated both in terms of magnitude and timing to ensure the efficient regeneration of muscle after injury (35). Unlike the traditional binary approach to describe macrophage polarization defined based on *in vitro* studies (100), our use of AIMs and ARMs includes the spectrum of phenotypes that lie between the two exaggerated M1 or M2 states. In physiological settings, macrophages manifest a variety of intermediate states where often the canonical markers of both M1 and M2 macrophages can be co-expressed in injured tissue (101). Further, following acute injury *in vivo*, macrophage profiles that do not align with either binary state, indicating broader macrophage plasticity along their path to polarization from pro-inflammatory to pro-regenerative states (33, 34, 101, 102). This diversity is also noted in several dysregulated microenvironments associated with lipid, disease, tumor, and scar, where macrophages show unique transcriptional profiles related to the specialized state of the tissue where they exist (103–106). Additionally, given the acute nature of the AIM and ARM states, there is a temporal element to their polarizations which contributes to the intermediate phenotypes as they transition along the progression from inflammatory to regenerative.

**Figure 4:**
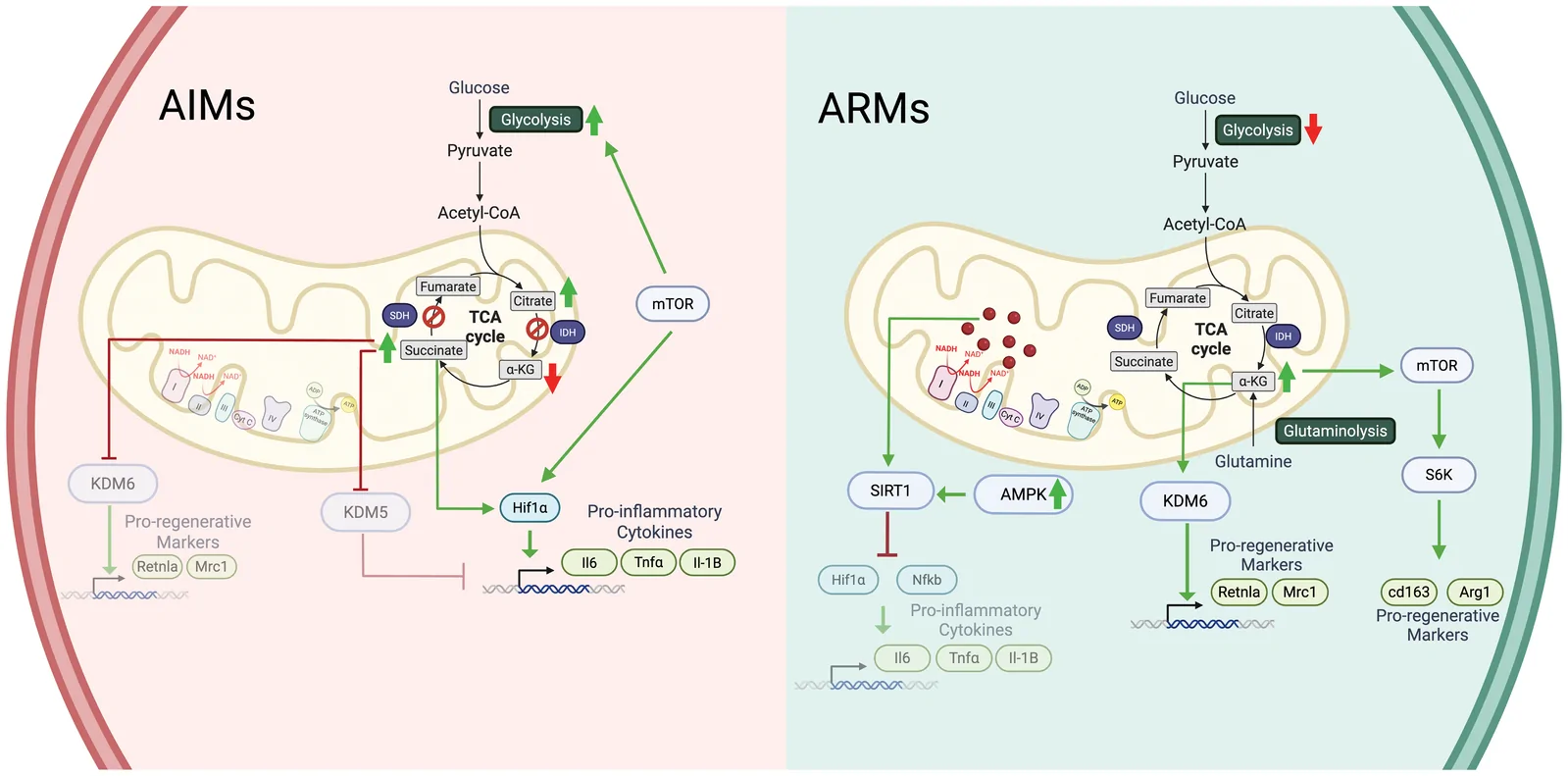
The metabolic epigenetic axis of macrophage found in acutely injured muscles. The AIMs, exhibit increased utilization of glycolysis, mTOR signaling, as well as an accumulation of succinate and citrate increasing stability of HIFα and inhibiting activity of lysine demethylases (KDMs), which supports the transcription of pro-inflammatory factors like *Tnf*α, *Il6* and *Il1b*. ARMs utilize OXPHOS and the TCA cycle, increasing accumulation of αKG leading to activation of KDMs increasing expression of *Retnla* and *Mrc1.* This also increases NAD+, activating SIRTs and lowering expression of pro-inflammatory factors like *Tnf*α, *Il6* and *Il1b*. Additionally, mTOR signaling in this case can prompt expression of pro-regenerative markers through S6K activation. Increased AMPK promotes Sirt activity as well, further inhibiting the expression of transcription factors *Hifα* and *Nfkb* as well as their downstream pro-inflammatory genes.

One of the molecular basis underlying diversity of macrophage polarization across the inflammatory and regenerative spectrum is metabolism, which impacts macrophage profile through various cellular metabolites, whose levels alter downstream epigenetic changes (15, 20, 107) (**Figures 1**, **4**). As metabolic flux can change continually and respond to environmental and internal cues, it allows for the plasticity of states along the gradient of AIMs and ARMs (**Figure 4**). During the early (inflammatory) state of the injured tissue, macrophages rely on glycolysis for energy, leading to increased production of reactive oxygen species (ROS) (108–110). Downstream impacts of this metabolic feature include IL-6 and IL-1B secretion via the ROS-PKM2-STAT3 axis (111, 112). Additionally, in AIMs the TCA cycle is disrupted due to downregulation of the gene encoding enzyme isocitrate dehydrogenase (IDH), leading to an accumulation of itaconate which inhibits succinate dehydrogenase (SDH) further driving accumulation of succinate (113, 114). The accumulation of these metabolites prevents degradation of HIF1α, a pro-inflammatory associated transcription factor that drives transcription of pro-inflammatory cytokines including *Il1β, Il6*, and *Tnfα* (115–117). Increased succinate accumulation has also been found to inhibit lysine demethylases (KDMs) via alpha-ketoglutarate (αKG) antagonization (118). One such regulator, KDM5, demethylates H3K4me3, which is a primary epigenetic marker for active gene transcription (119–121). Therefore, limiting the activity of KDM5, helps maintain H3K4me3 at the promoters of pro-inflammatory cytokines like *Il6* and *Tnfα* to allow their continued expression (122). The Akt-mTOR-HIF1α axis also plays a role in macrophage activation, acting as a master regulator of glucose metabolism. mTOR signaling, specifically in the context of the mTORC1 protein complex, increases HIF1α activation which in turn increases glycolytic activity and the expression of its downstream pro-inflammatory cytokines (123–127) (**Figure 4**).

As the reparative process progresses, macrophages transition into ARMs, where they rely on oxidative phosphorylation (OXPHOS) as the primary source of energy (108, 109, 128). Additionally, while not essential for their pro-regenerative polarization, these macrophages increase fatty acid oxidation utilization (129–131). Unlike AIMs, the TCA cycle remains intact in ARMs, and its function, along with glutaminolysis, leads to accumulation of αKG in these macrophages (132–134). αKG activates KDMs, such as KDM6, which removes the repressive methylation mark H3K27me3 leading to increased expression of pro-regenerative genes like *Retnla*, *Mrc1*, and *Il6*, including in macrophages (134–138). Another cofactor produced during OXPHOS that links metabolism and epigenetics is NAD+, which is more abundant in ARMs than AIMs (139). NAD+ is a cofactor for Sirtuins (Sirt) – a protein modifying enzyme family with various epigenetic modifying functions (140, 141). Sirt1 alters gene expression at the epigenetic level by histone modifications which impact chromatin accessibility, and at the post-translational level through its protein deacetylation activity. Sirt1 activity is largely dependent on NAD+ levels with higher activity promoting a pro-regenerative macrophage phenotype (142). Another metabolic signal that supports the transition from AIMs to ARMs is AMPKα1 (14, 143). Increased Sirt1 activity by NAD+ helps repress inflammatory transcription factors HIF1α and NFKB1 (143). This in turn promotes pro-regenerative polarization of macrophages by AMPKα signaling, reducing chronic inflammatory response and supporting SC differentiation in muscle (142–146). This protein kinase acts as an energy sensor, activating (and preserving) OXPHOS to promote ATP synthesis (147, 148). As a result, AMPK activates Sirt1 through accumulation of NAD+, but also by increasing Sirt1 expression directly, contributing to the pro-regenerative fate of the ARMs (149–151) (**Figure 4**). Although mTOR signaling supports AIM associated transcriptional pathways, mTOR can also affect pro-regenerative polarization through the Akt-mTOR-S6Kinase axis in the presence of Substance-P or increased αKG (152, 153). However, mTOR signaling impacts a variety of downstream targets, allowing its context-dependent actions that promote AIM or ARM states (124).

### Dysregulated macrophage polarization and lack of inflammation resolution in muscle disease

During muscle repair, macrophages transition between a spectrum of AIM to ARM states through metabolic variation and local inflammatory cues. A break in the connection between metabolism and gene regulation can shift the niche into pathogenic states and prevent the normal progression of the tissue reparative process. Such dysregulations are associated with a variety of chronic inflammatory conditions including atherosclerosis, diabetes, rheumatoid arthritis, Alzheimer’s disease, and muscular dystrophies (154–158).

Muscular dystrophy is characterized by a propensity for excessive muscle damage, often accompanied by diminished repair capacity and progressive degeneration, leading to persistent, unresolved inflammation with sustained pro-inflammatory cytokine and chemokine signaling (40, 155–160). These deficits prevent the tissue from regenerating efficiently and are associated with prolonged presence of neutrophils and macrophages, which can further damage healthy muscle tissue, limit subsequent regeneration, and exacerbate fibrotic and adipogenic replacement of muscle (**Figure 2B**) (6, 22, 23, 31, 161, 162).

**Figure 5:**
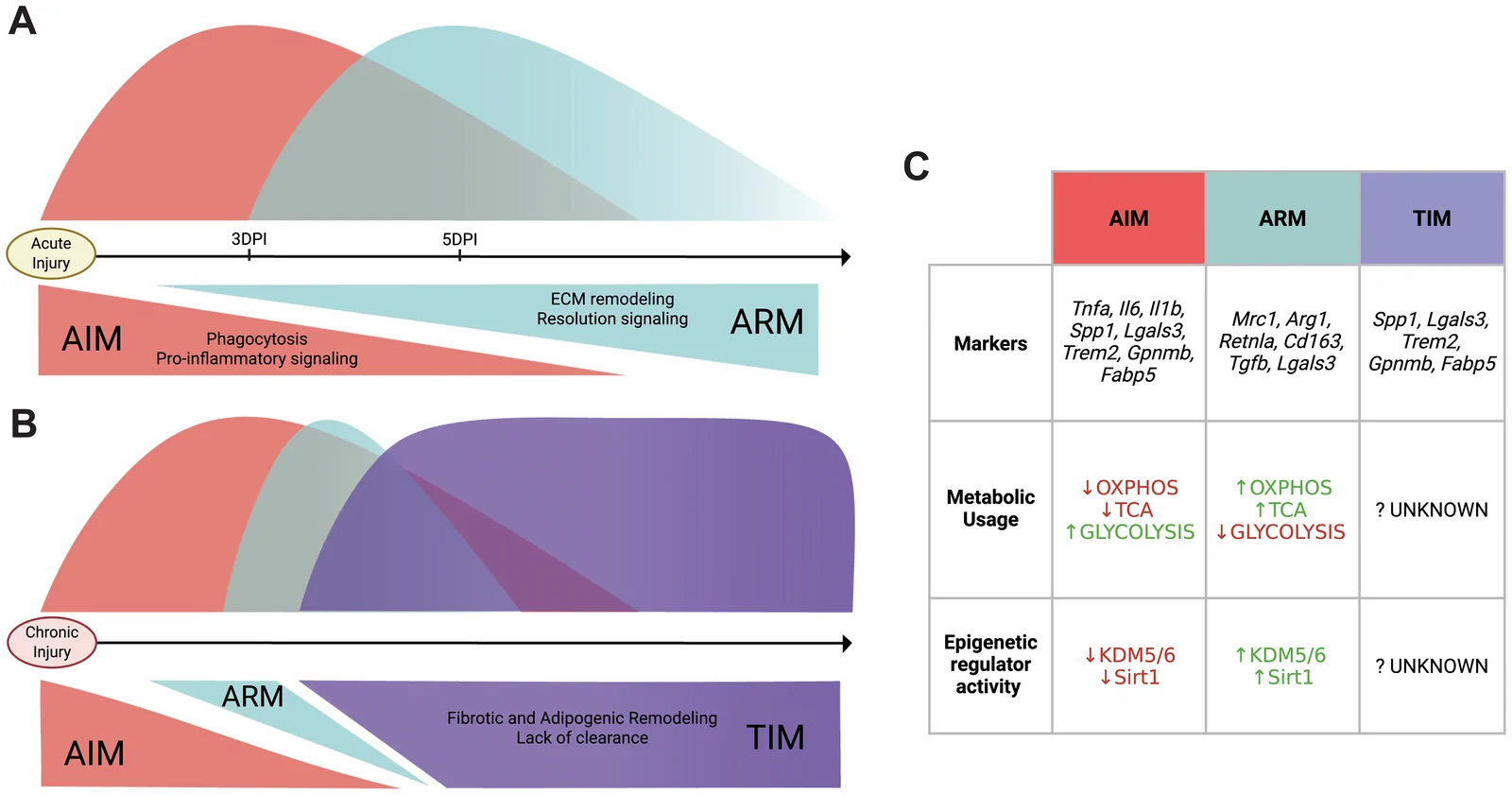
Temporal and molecular features of macrophages in injured muscles. **(A)** Following an acute muscle injury, infiltrating monocytes adopt an acute inflammatory macrophage (AIM) phenotype, which peaks around 3 days post injury. Then this phenotypic signature fades, as AIMs adopt an acute regenerative macrophage (ARM) phenotype, through intermediate functional phenotypes. The ARMs become the dominant macrophage around 5 days post injury and their phenotypic signature peaks before the resolution of inflammation. **(B)** In chronically inflamed tissue, AIMs fail to resolve and give rise to TIMs which not only persist for an extended period in the tissue but also impair muscle regeneration. **(C)** Aside from their temporal features, these macrophages also exhibit molecular, metabolic and epigenetic features summarized in the table. Gene expression markers of AIMs include *Tnfa, Il6, Il1b, Spp1, Lgals3, Trem2, Gpnmb*, and *Fabp5* (16, 111, 115, 162, 164–167). Markers of ARMs include *Mrc1, Arg1, Retnla, Cd163, Tgfb*, and *Lgals3* (16, 134, 136, 152, 166, 168). Markers of TIMs include *Spp1, Lgals3, Trem2, Gpnmb*, and *Fabp5* (47–50).

Recent single cell, single nuclei, and spatial transcriptomic and proteomic studies have identified disease-specific macrophages across muscular dystrophies and myopathies missing from the healthy muscles (18, 47–49, 92, 162, 163). Characterization of the transcriptional profile of these cells from models of varying disease severity, ranging from models of LAMA2 congenital muscular dystrophy to Duchenne muscular dystrophy, have identified similar macrophage population enriched in disease conditions (47, 49, 50). Here, instead of inflammation being resolved in a timely manner, as is the case in acute injury scenarios (**Figure 5**), these disease-associated (pathogenic) macrophages persist and progressively accumulate within the tissue with disease progression, leading to chronically inflamed tissue. In this chronic inflammatory state, inflammatory signaling by TIMs prevents the tissue from returning to its homeostatic state (**Figure 5**). These pathogenic macrophages are detected not only in chronically inflamed muscles, but also in chronically inflamed and pro-fibrotic brain, liver, lungs, and adipose tissues, and are associated with tumors or enriched during aging (103, 169–177). This highlights the broad relevance of TIMs across tissues and disease conditions, as well as the need to better understand their origin and fate. These cells have been shown to arise from both monocytes recruited to damaged tissue, as well as from tissue resident macrophages, suggesting that their origin is associated with signals stemming from within the tissue micro-environment, rather than an innate feature of macrophages or their progenitors (58).

Importantly, TIMs are characterized by heightened expression of *Spp1*, *Lgals3*, *Trem2*, *Fabp5* and *Gpnmb* regardless of condition (47–50). Many of these markers upregulated in TIMs are known to contribute to fibrotic or adipogenic signaling through their impact on neighboring cells within the niche. Macrophages expressing *Spp1* interact with muscle fibroblasts and drive fibrosis through heightened secretion of osteopontin, fibronectin, and semaphorins (178–180). This is the case in multiple disease conditions throughout various tissues where macrophages with high *Spp1* expression contribute to fibrotic deposition in lung, liver, heart, endometrium, kidney, skin and dystrophic muscle (20, 148, 156–158). Expression of *Trem2* has also been associated with fibrosis especially in the context of pulmonary diseases, although in other tissues the connection to fibrosis is not clear, and depending on the context, can confer both protective or pathogenic attributes (181–185). In addition, some of the markers associated with TIMs, for instance *Lgals3, Spp1*, and *Trem2*, are also known to play a role in promoting adipogenesis and fat accumulation in disease (186–188).

Interestingly however, many of the genes associated with TIMs are transiently expressed by AIMs or ARMs during the acute muscle repair process (**Figure 5**). For example, macrophages present in a muscle 3 days post injury (AIMs), transiently express *Spp1* and *Trem2*, similar to macrophages in acutely injured spinal cord, but these macrophages transiently expressing *Spp1* and *Trem2* do not cause fibroadipogenic tissue degeneration (162, 164, 181, 189). Similarly, *Gpnmb* and *Spp1* expressing macrophages are involved in lung repair processes, and it is suggested that their propensity to induce fibrosis is a vestige of a temporal mechanism of their appearance and clearance, not their intrinsic feature (165). *Lgals3*, another TIM marker, is increased in AIMs and ARMs, indicating that acute *Lgals3* expression is not inherently pathogenic (166, 168). Other *in vitro* studies show *Gpnmb* and *Fapb5* increase upon pro-inflammatory stimulation with LPS or IFNγ, with *Gpnmb* associated with a negative feedback loop for pro-inflammatory signaling, while *Fabp5* deletion promotes transition towards a pro-regenerative phenotype (167, 190). The transient expression of these TIM-enriched genes thus seems to play a role in in a properly regulated acute inflammatory response. This further supports the notion that environmental factors in diseased tissue underlie this inability of TIMs to exit a pro-inflammatory state in disease conditions.

One potential mechanism by which the diseased tissue niche may push macrophages toward the TIM state is through dysregulation of the metabolic–epigenetic axis driven by the aberrant metabolic signature of chronically inflamed tissues (47, 142, 191). Such metabolic disturbances may interfere with epigenetic programs required for gene expression associated with inflammation resolution. For example, *Trem2*-expressing macrophages in diseased tissues exhibit altered lipid metabolism and related changes that influence metabolite accumulation, thereby reshaping the macrophage epigenetic landscape (191). TIMs also show increased expression of *Fabp5*, which limits fatty acid accumulation and restricts TCA cycle activity and OXPHOS, which may ultimately restrict their timely transition to ARMs (192). Although the expression of these signature genes is not limited to TIMs, their combined and prolonged expression likely promotes extended macrophage accumulation and pathogenic interactions within the stroma of the diseased tissue niche. The interplay between environmental cues and the metabolic–epigenetic profile of TIMs remains an active area of investigation, and defining the full complement of factors that prevent inflammation resolution is critical to clarify how metabolism contributes to TIMs in disease and whether these cells can be therapeutically redirected toward a more AIM-like phenotype for efficient resolution.

### Therapeutic approaches to target dysregulated inflammatory responses in muscle disease

Therapeutic endeavors for chronic inflammatory myopathies, as well as other inflammatory diseases, have primarily focused on the suppression of inflammation. For instance, the use of anti-inflammatory glucocorticoids (GC) is the standard of care for pediatric DMD patients as it improves patients’ longevity and quality of life by limiting inflammation and associated pathologies (193–195). Despite the positive impact of GC therapy, their long term use is associated with a variety of serious adverse effects, and can even increase muscle wasting and weakness over time (196, 197). In fact, broad ablation of inflammatory processes is detrimental to myogenic capacity in dystrophic muscle, where spontaneous damage continues to occur frequently with disease course (198). Although GCs effectively inhibit macrophage polarization toward AIMs, in diseases dominated by tissue inflammation, the use of GCs can also suppress the expression of ARM markers (199) further limiting repair processes. As acute inflammation is critical for efficient muscle regeneration and a return to homeostasis after injury, broad therapeutic inhibition may not be as beneficial as promoting resolution of chronic inflammation in disease. This is supported by the recognition that TIMs are transcriptionally similar to AIMs, but temporally dysregulated as they fail to clear following tissue injury (**Figure 5**).

A therapeutic approach to target TIMs and stem the impact of chronic inflammation is termed “Resolution Pharmacology”, which leverages existing resolution pathways to clear inflammation and promote repair (200). Such pro-resolution pathways include Resolvins and the Anxa1-FPR2 signaling axis which is involved in regulation of the initial inflammatory signaling of neutrophils and also the timely clearance of both neutrophils and pro-inflammatory macrophages after injury (200–203). AnxA1 signaling has been implicated not just in the appearance of ARMs but plays a role throughout the entire inflammatory process. For example, in the absence of Anxa1, there is reduced inflammatory signals like IL-1β and TNF-α upon LPS stimulation, indicating the necessity for Anxa1 in both the AIM and ARM states (204). Pro-resolving therapies, including small molecule and mimetic peptide agonists of FPR2, have been shown to have beneficial effects following myocardial infarction and on disease pathogenesis in dystrophic cardiac and skeletal muscle, where it effectively cleared inflammation and promoted enhanced repair (143, 205, 206). Additionally, use of the pro-resolving mediator Resolvin D1 showed regenerative improvements following healthy skeletal muscle injury (207). Thus, targeting resolution of inflammation, rather than blunting inflammation altogether, may be the key to balancing the benefits of acute inflammation while preventing the pathogenic effects of chronic TIM persistence.

### Metabolic and epigenetic impacts of therapeutic interventions for muscle disease

Treatment with GCs impacts the metabolic-epigenetic axis of macrophages *in vitro*, such that dexamethasone represses glycolytic usage in macrophages polarized towards AIMs, but also represses mitochondrial respiration of macrophages polarized towards ARMs (208). GCs mitigate inflammatory response of macrophages by promoting TCA cycle flux, thus preventing accumulation of succinate which is a known stabilizer of pro-inflammatory transcription factor HIF1α (209–211). Additionally, GCs have been shown to decrease glycolytic activity of pro-inflammatory macrophages in culture (210). These findings show that treatment with GC can shift the metabolic profile of macrophages away from a pro-inflammatory phenotype.

Pro-resolving therapies also impact the metabolic-epigenetic axis of macrophages. One such therapy that targets the AnxA1-FPR2 axis, promotes macrophage transition from AIMs to ARMs in part by with altering their metabolism via AMPK signaling (143). As previously described, AMPK is known to increase OXPHOS activity and promote Sirt activity. ANXA1-FPR2 binding activates AMPK, connecting this pro-resolution therapy to mechanisms of metabolic and epigenetic regulation of macrophage phenotype (143). Unlike pro-resolving therapies that modulates AMPK activity, GC therapies do not appear to regulate AMPK activity itself (212).

Given the ability of the ANXA1-FPR2 axis to alter the metabolic-epigenetic axis of macrophages through AMPK signaling and Sirt1 activity, an additional approach to promote resolution of inflammation is activating Sirt1 either directly through small molecules or by increasing NAD+ availability to trigger the transition towards ARMs (213–215). This approach has been attempted in a variety of inflammatory diseases and has shown promise in disease models and in pre-clinical trials in dampening the inflammatory response in patients (216–218). Additional testing is needed to confirm the efficacy and specificity of the SIRT1-targeting drugs and develop more potent and specific options to combat longer term inflammation without negative effects on the rest of the system (219).

## Discussion

Macrophages in skeletal muscle are dynamic regulators of repair whose behavior emerges from integrated metabolic and epigenetic programs rather than fixed categories. By connecting acute injury responses with chronic disease pathology, we highlight macrophage plasticity as a central requirement for successful regeneration and identify loss of this plasticity as a key feature of persistent inflammation and fibrosis in disease. A major implication of this mode of macrophage action is the need to move beyond marker-based classification toward a continuum-based model in which functional states are linked to metabolic and other molecular transitions. This includes the shift between glycolysis and oxidative phosphorylation being the spectrum that guides epigenetic regulation of inflammatory and regenerative gene programs. This understanding supports greater use of multi-omics and spatial technologies to track macrophage states over time and across tissue niches, with particular focus on how environmental cues drive divergence into resolving or pathogenic trajectories. This is especially relevant in chronic diseases such as muscular dystrophies where these trajectories are stalled or disrupted. Such a framework supports efforts to define disease-associated macrophage populations that arise from disrupted metabolic and epigenetic control and contribute to failed regeneration. Understanding how these states are maintained may guide strategies to interrupt maladaptive signaling loops that sustain inflammation and fibrosis by shifting therapeutic direction from broad immunosuppression toward interventions that restore macrophage plasticity and promote inflammation resolution. Targeting metabolic and epigenetic pathways is therefore positioned as a promising avenue for reestablishing tissue regenerative capacity in muscle and related chronic inflammatory diseases.

## References

[1] Medzhitov R . Origin and physiological roles of inflammation. Nature. (2008) 454:428–35. doi: 10.1038/nature07201 18650913

[2] Janssen WJ Henson PM . Cellular regulation of the inflammatory response. Toxicol Pathol. (2012) 40:166–73. doi: 10.1177/0192623311428477 22089838 PMC4138834

[3] Mason JC Libby P . Cardiovascular disease in patients with chronic inflammation: mechanisms underlying premature cardiovascular events in rheumatologic conditions. Eur Heart J. (2015) 36:482–489c. doi: 10.1093/eurheartj/ehu403 25433021 PMC4340364

[4] Porter JD Khanna S Kaminski HJ Rao JS Merriam AP Richmonds CR . A chronic inflammatory response dominates the skeletal muscle molecular signature in dystrophin-deficient mdx mice. Hum Mol Genet. (2002) 11:263–72. doi: 10.1093/hmg/11.3.263 11823445

[5] Chavda VP Feehan J Apostolopoulos V . Inflammation: The cause of all diseases. Cells. (2024) 13:1906. doi: 10.3390/cells13221906 39594654 PMC11592557

[6] Tu H Li YL . Inflammation balance in skeletal muscle damage and repair. Front Immunol. (2023) 14. doi: 10.3389/fimmu.2023.1133355 36776867 PMC9909416

[7] Peake JM Neubauer O Della Gatta PA Nosaka K . Muscle damage and inflammation during recovery from exercise. J Appl Physiol (1985). (2017) 122:559–70. doi: 10.1152/japplphysiol.00971.2016 28035017

[8] Chazaud B Brigitte M Yacoub-Youssef H Arnold L Gherardi R Sonnet C . Dual and beneficial roles of macrophages during skeletal muscle regeneration. Exerc Sport Sci Rev. (2009) 37:18–22. doi: 10.1097/JES.0b013e318190ebdb 19098520

[9] Yang W Hu P . Skeletal muscle regeneration is modulated by inflammation. J Orthopaedic Translation. (2018) 13:25–32. doi: 10.1016/j.jot.2018.01.002 29662788 PMC5892385

[10] Tidball JG . Regulation of muscle growth and regeneration by the immune system. Nat Rev Immunol. (2017) 17:165–78. doi: 10.1038/nri.2016.150 28163303 PMC5452982

[11] Chazaud B Sonnet C Lafuste P Bassez G Rimaniol AC Poron F . Satellite cells attract monocytes and use macrophages as a support to escape apoptosis and enhance muscle growth. J Cell Biol. (2003) 163:1133–43. doi: 10.1083/jcb.200212046 14662751 PMC2173611

[12] Tidball JG . Inflammatory processes in muscle injury and repair. Am J Physiol Regul Intgr Comp Physiol. (2005) 288:R345–53. doi: 10.1152/ajpregu.00454.2004 15637171

[13] Saclier M Cuvellier S Magnan M Mounier R Chazaud B . Monocyte/macrophage interactions with myogenic precursor cells during skeletal muscle regeneration. FEBS J. (2013) 280:4118–30. doi: 10.1111/febs.12166 23384231

[14] Mounier R Théret M Arnold L Cuvellier S Bultot L Göransson O . AMPKα1 regulates macrophage skewing at the time of resolution of inflammation during skeletal muscle regeneration. Cell Metab. (2013) 18:251–64. doi: 10.1016/j.cmet.2013.06.017 23931756

[15] Thapa B Lee K . Metabolic influence on macrophage polarization and pathogenesis. BMB Rep. (2019) 52:360–72. doi: 10.5483/BMBRep.2019.52.6.140 31186085 PMC6605523

[16] Arnold L Henry A Poron F Baba-Amer Y van Rooijen N Plonquet A . Inflammatory monocytes recruited after skeletal muscle injury switch into antiinflammatory macrophages to support myogenesis. J Exp Med. (2007) 204:1057–69. doi: 10.1084/jem.20070075 17485518 PMC2118577

[17] Li R Li X Zhang X Yu J Li Y Ran S . Macrophages in cardiovascular fibrosis: Novel subpopulations, molecular mechanisms, and therapeutic targets. Can J Cardiol. (2025) 41:309–22. doi: 10.1016/j.cjca.2024.11.018 39580052

[18] Borok M Didier N Gattazzo F Ozturk T Corneau A Rouard H . Progressive and coordinated mobilization of the skeletal muscle niche throughout tissue repair revealed by single-cell proteomic analysis. Cells. (2021) 10:744. doi: 10.3390/cells10040744 33800595 PMC8066646

[19] Morgan J Partridge T . Skeletal muscle in health and disease. Dis Model Mech. (2020) 13:dmm042192. doi: 10.1242/dmm.042192 32066552 PMC7044447

[20] Caballero-Sánchez N Alonso-Alonso S Nagy L . Regenerative inflammation: When immune cells help to re-build tissues. FEBS J. (2024) 291:1597–614. doi: 10.1111/febs.16693 36440547 PMC10225019

[21] Hogarth MW Kurukunda MP Ismat K Uapinyoying P Jaiswal JK . Exploring the therapeutic potential of fibroadipogenic progenitors in muscle disease. J Neuromuscul Dis. (2025) 12:3–14. doi: 10.1177/22143602241298545 39973455 PMC11949306

[22] St. Pierre Schneider B Brickson S Corr DT Best T . CD11b+ neutrophils predominate over RAM11+ macrophages in stretch-injured muscle. Muscle Nerve. (2002) 25:837–44. doi: 10.1002/mus.10109 12115972

[23] Teixeira CFP Zamunér SR Zuliani JP Fernandes CM Cruz-Hofling MA Fernandes I . Neutrophils do not contribute to local tissue damage, but play a key role in skeletal muscle regeneration, in mice injected with Bothrops asper snake venom. Muscle Nerve. (2003) 28:449–59. doi: 10.1002/mus.10453 14506717

[24] Wang J Hossain M Thanabalasuriar A Gunzer M Meininger C Kubes P . Visualizing the function and fate of neutrophils in sterile injury and repair. Science. (2017) 358:111–6. doi: 10.1126/science.aam9690 28983053

[25] Torres-Ruiz J Alcalá-Carmona B Alejandre-Aguilar R Gómez-Martín D . Inflammatory myopathies and beyond: The dual role of neutrophils in muscle damage and regeneration. Front Immunol. (2023) 14:1113214. doi: 10.3389/fimmu.2023.1113214 36923415 PMC10008923

[26] Gong Y Koh DR . Neutrophils promote inflammatory angiogenesis via release of preformed VEGF in an *in vivo* corneal model. Cell Tissue Res. (2010) 339:437–48. doi: 10.1007/s00441-009-0908-5 20012648

[27] Kaplanski G Marin V Montero-Julian F Mantovani A Farnarier C . IL-6: a regulator of the transition from neutrophil to monocyte recruitment during inflammation. Trends Immunol. (2003) 24:25–9. doi: 10.1016/S1471-4906(02)00013-3 12495721

[28] Sun D Martinez CO Ochoa O Ruiz-Willhite L Bonilla JR Centonze VE . Bone marrow-derived cell regulation of skeletal muscle regeneration. FASEB J. (2009) 23:382–95. doi: 10.1096/fj.07-095901 18827026 PMC2630778

[29] Lu H Huang D Ransohoff RM Zhou L . Acute skeletal muscle injury: CCL2 expression by both monocytes and injured muscle is required for repair. FASEB J. (2011) 25:3344–55. doi: 10.1096/fj.10-178939 21697550 PMC3177578

[30] Torfs K Vermeersch G Gouwy M Devos T Proost P Struyf S . Neutrophils as critical orchestrators of chronic inflammation. Cell Mol Immunol. (2026) 23:123–49. doi: 10.1038/s41423-025-01380-w 41530536 PMC12858905

[31] Tulangekar A Sztal TE . Inflammation in Duchenne muscular dystrophy–exploring the role of neutrophils in muscle damage and regeneration. Biomedicines. (2021) 9:1366. doi: 10.3390/biomedicines9101366 34680483 PMC8533596

[32] Geissmann F Jung S Littman DR . Blood monocytes consist of two principal subsets with distinct migratory properties. Immunity. (2003) 19:71–82. doi: 10.1016/S1074-7613(03)00174-2 12871640

[33] Varga T Mounier R Horvath A Cuvellier S Dumont F Poliska S . Highly dynamic transcriptional signature of distinct macrophage subsets during sterile inflammation, resolution, and tissue repair. J Immunol. (2016) 196:4771–82. doi: 10.4049/jimmunol.1502490 27183604

[34] Witherel CE Sao K Brisson BK Han B Volk SW Petrie RJ . Regulation of extracellular matrix assembly and structure by hybrid M1/M2 macrophages. Biomaterials. (2021) 269:120667. doi: 10.1016/j.biomaterials.2021.120667 33450585 PMC7870567

[35] Wang X Zhou L . The many roles of macrophages in skeletal muscle injury and repair. Front Cell Dev Biol. (2022) 10:952249. doi: 10.3389/fcell.2022.952249 35898401 PMC9309511

[36] Tidball JG Villalta SA . Regulatory interactions between muscle and the immune system during muscle regeneration. Am J Physiol Regul Integr Comp Physiol. (2010) 298:R1173–87. doi: 10.1152/ajpregu.00735.2009 20219869 PMC2867520

[37] Tidball JG . Mechanisms of muscle injury, repair, and regeneration. Compr Physiol. (2011) 1:2029–62. doi: 10.1002/j.2040-4603.2011.tb00387.x 23733696

[38] Ziemkiewicz N Hilliard G Pullen NA Garg K . The role of innate and adaptive immune cells in skeletal muscle regeneration. Int J Mol Sci. (2021) 22:3265. doi: 10.3390/ijms22063265 33806895 PMC8005179

[39] Juhas M Abutaleb N Wang JT Ye J Shaikh Z Sriworarat C . Incorporation of macrophages into engineered skeletal muscle enables enhanced muscle regeneration. Nat BioMed Eng. (2018) 2:942–54. doi: 10.1038/s41551-018-0290-2 30581652 PMC6296488

[40] Hernandez-Torres F Matias-Valiente L Alzas-Gomez V Aranega AE . Macrophages in the context of muscle regeneration and Duchenne muscular dystrophy. Int J Mol Sci. (2024) 25:10393. doi: 10.3390/ijms251910393 39408722 PMC11477283

[41] Zhang J Qu C Li T Cui W Wang X Du J . Phagocytosis mediated by scavenger receptor class BI promotes macrophage transition during skeletal muscle regeneration. J Biol Chem. (2019) 294:15672–85. doi: 10.1074/jbc.RA119.008795 31462534 PMC6816089

[42] Perdiguero E Sousa-Victor P Ruiz-Bonilla V Jardí M Caelles C Serrano AL . p38/MKP-1–regulated AKT coordinates macrophage transitions and resolution of inflammation during tissue repair. J Cell Biol. (2011) 195:307–22. doi: 10.1083/jcb.201104053 21987635 PMC3198158

[43] Baht GS Bareja A Lee DE Rao RR Huang R Huebner JL . Meteorin-like facilitates skeletal muscle repair through a Stat3/IGF-1 mechanism. Nat Metab. (2020) 2:278–89. doi: 10.1038/s42255-020-0184-y 32694780 PMC7504545

[44] Mantovani A Sozzani S Locati M Allavena P Sica A . Macrophage polarization: tumor-associated macrophages as a paradigm for polarized M2 mononuclear phagocytes. Trends Immunol. (2002) 23:549–55. doi: 10.1016/S1471-4906(02)02302-5 12401408

[45] Dort J Fabre P Molina T Dumont NA . Macrophages are key regulators of stem cells during skeletal muscle regeneration and diseases. Stem Cells Int. (2019) 2019:4761427. doi: 10.1155/2019/4761427 31396285 PMC6664695

[46] Serhan CN Savill J . Resolution of inflammation: the beginning programs the end. Nat Immunol. (2005) 6:1191–7. doi: 10.1038/ni1276 16369558

[47] Coulis G Jaime D Guerrero-Juarez C Kastenschmidt JM Farahat PK Nguyen Q . Single-cell and spatial transcriptomics identify a macrophage population associated with skeletal muscle fibrosis. Sci Adv. (2023) 9:eadd9984. doi: 10.1126/sciadv.add9984 37418531 PMC10328414

[48] Wang X Moy JK Wang Y Smith GR Ruf-Zamojski F Przytycki PF . Heterogeneous macrophage activation in acute skeletal muscle sterile injury and mdx5cv model of muscular dystrophy. Int J Mol Sci. (2025) 26:8098. doi: 10.3390/ijms26168098 40869418 PMC12387070

[49] Patsalos A Halasz L Oleksak D Wei X Nagy G Tzerpos P . Spatiotemporal transcriptomic mapping of regenerative inflammation in skeletal muscle reveals a dynamic multilayered tissue architecture. J Clin Invest. (2024) 134:e173858. doi: 10.1172/JCI173858 39190487 PMC11473166

[50] de Menezes YKT Lee J Cheng-Zhang JQ Johnson MA Ranatunga RN Kemaladewi DU . Targeting Galectin-3 to modulate inflammation in LAMA2-deficient congenital muscular dystrophy. bioRxiv. (2025), 2025.03.12.642905. doi: 10.1101/2025.03.12.642905 40161708 PMC11952532

[51] Uderhardt S Martins AJ Tsang JS Lämmermann T Germain RN . Resident macrophages cloak tissue microlesions to prevent neutrophil-driven inflammatory damage. Cell. (2019) 177:541–555.e17. doi: 10.1016/j.cell.2019.02.028 30955887 PMC6474841

[52] Cailhier JF Partolina M Vuthoori S Wu S Ko K Watson S . Conditional macrophage ablation demonstrates that resident macrophages initiate acute peritoneal inflammation1. J Immunol. (2005) 174:2336–42. doi: 10.4049/jimmunol.174.4.2336 15699170

[53] Wang X Sathe AA Smith GR Ruf-Zamojski F Nair V Lavine KJ . Heterogeneous origins and functions of mouse skeletal muscle-resident macrophages. Proc Natl Acad Sci. (2020) 117:20729–40. doi: 10.1073/pnas.1915950117 32796104 PMC7456122

[54] Babaeijandaghi F Cheng R Kajabadi N Soliman H Chang CK Smandych J . Metabolic reprogramming of skeletal muscle by resident macrophages points to CSF1R inhibitors as muscular dystrophy therapeutics. Sci Transl Med. (2022) 14:eabg7504. doi: 10.1126/scitranslmed.abg7504 35767650

[55] Babaeijandaghi F Kajabadi N Long R Tung LW Cheung CW Ritso M . DPPIV+ fibro-adipogenic progenitors form the niche of adult skeletal muscle self-renewing resident macrophages. Nat Commun. (2023) 14:8273. doi: 10.1038/s41467-023-43579-3 38092736 PMC10719395

[56] Nobs SP Kopf M . Tissue-resident macrophages: guardians of organ homeostasis. Trends Immunol. (2021) 42:495–507. doi: 10.1016/j.it.2021.04.007 33972166

[57] Wang X Zhou L . The multifaceted role of macrophages in homeostatic and injured skeletal muscle. Front Immunol. (2023) 14:1274816. doi: 10.3389/fimmu.2023.1274816 37954602 PMC10634307

[58] Wang Y Wang X Alabdullatif S Homma ST Alekseyev YO Zhou L . Expansion and pathogenic activation of skeletal muscle–resident macrophages in mdx5cv/Ccr2-/- mice. Proc Natl Acad Sci USA. (2025) 122:e2410095122. doi: 10.1073/pnas.2410095122 40067893 PMC11929395

[59] Brigitte M Schilte C Plonquet A Baba-Amer Y Henri A Charlier C . Muscle resident macrophages control the immune cell reaction in a mouse model of notexin-induced myoinjury. Arthritis Rheumatism. (2010) 62:268–79. doi: 10.1002/art.27183 20039420

[60] Juban G Saclier M Yacoub-Youssef H Kernou A Arnold L Boisson C . AMPK activation regulates LTBP4-dependent TGF-β1 secretion by pro-inflammatory macrophages and controls fibrosis in Duchenne muscular dystrophy. Cell Rep. (2018) 25:2163–2176.e6. doi: 10.1016/j.celrep.2018.10.077 30463013

[61] Lemos DR Babaeijandaghi F Low M Chang CK Lee ST Fiore D . Nilotinib reduces muscle fibrosis in chronic muscle injury by promoting TNF-mediated apoptosis of fibro/adipogenic progenitors. Nat Med. (2015) 21:786–94. doi: 10.1038/nm.3869 26053624

[62] Zhang C Li Y Wu Y Wang L Wang X Du J . Interleukin-6/Signal transducer and activator of transcription 3 (STAT3) pathway is essential for macrophage infiltration and myoblast proliferation during muscle regeneration. J Biol Chem. (2013) 288:1489–99. doi: 10.1074/jbc.M112.419788 23184935 PMC3548462

[63] Forcina L Miano C Musarò A . The physiopathologic interplay between stem cells and tissue niche in muscle regeneration and the role of IL-6 on muscle homeostasis and diseases. Cytokine Growth Factor Rev. (2018) 41:1–9. doi: 10.1016/j.cytogfr.2018.05.001 29778303

[64] Forcina L Franceschi C Musarò A . The hormetic and hermetic role of IL-6. Ageing Res Rev. (2022) 80:101697. doi: 10.1016/j.arr.2022.101697 35850167

[65] Horsley V Jansen KM Mills ST Pavlath GK . IL-4 acts as a myoblast recruitment factor during mammalian muscle growth. Cell. (2003) 113:483–94. doi: 10.1016/S0092-8674(03)00319-2 12757709

[66] Dumont N Frenette J . Macrophages protect against muscle atrophy and promote muscle recovery *in vivo* and *in vitro*. Am J Pathol. (2010) 176:2228–35. doi: 10.2353/ajpath.2010.090884 20304951 PMC2861088

[67] Saclier M Yacoub-Youssef H Mackey AL Arnold L Ardjoune H Magnan M . Differentially activated macrophages orchestrate myogenic precursor cell fate during human skeletal muscle regeneration. Stem Cells. (2013) 31:384–96. doi: 10.1002/stem.1288 23169615

[68] Verma M Asakura Y Murakonda BSR Pengo T Latroche C Chazaud B . Muscle satellite cell cross-talk with a vascular niche maintains quiescence via VEGF and Notch signaling. Cell Stem Cell. (2018) 23:530–543.e9. doi: 10.1016/j.stem.2018.09.007 30290177 PMC6178221

[69] Joe AWB Yi L Natarajan A Le Grand F So L Wang J . Muscle injury activates resident fibro/adipogenic progenitors that facilitate myogenesis. Nat Cell Biol. (2010) 12:153–63. doi: 10.1038/ncb2015 20081841 PMC4580288

[70] Uezumi A Ito T Morikawa D Shimizu N Yoneda T Segawa M . Fibrosis and adipogenesis originate from a common mesenchymal progenitor in skeletal muscle. J Cell Sci. (2011) 124:3654–64. doi: 10.1242/jcs.086629 22045730

[71] Uezumi A Fukada S Yamamoto N Takeda S Tsuchida K . Mesenchymal progenitors distinct from satellite cells contribute to ectopic fat cell formation in skeletal muscle. Nat Cell Biol. (2010) 12:143–52. doi: 10.1038/ncb2014 20081842

[72] Kopinke D Roberson EC Reiter JF . Ciliary Hedgehog signaling restricts injury-induced adipogenesis. Cell. (2017) 170:340–351.e12. doi: 10.1016/j.cell.2017.06.035 28709001 PMC5617351

[73] Heredia JE Mukundan L Chen FM Mueller AA Deo RC Locksley RM . Type 2 innate signals stimulate fibro/adipogenic progenitors to facilitate muscle regeneration. Cell. (2013) 153:376–88. doi: 10.1016/j.cell.2013.02.053 23582327 PMC3663598

[74] Brorson J Lin L Wang J Bæk A Billeskov TB Thybo FF . Complementing muscle regeneration-fibro-adipogenic progenitor and macrophage-mediated repair of elderly human skeletal muscle. Nat Commun. (2025) 16:5233. doi: 10.1038/s41467-025-60627-2 40473693 PMC12141666

[75] Nawaz A Bilal M Fujisaka S Kado T Aslam MR Ahmed S . Depletion of CD206+ M2-like macrophages induces fibro-adipogenic progenitors activation and muscle regeneration. Nat Commun. (2022) 13:7058. doi: 10.1038/s41467-022-34191-y 36411280 PMC9678897

[76] Wosczyna MN Konishi CT Perez Carbajal EE Wang TT Walsh RA Gan Q . Mesenchymal stromal cells are required for regeneration and homeostatic maintenance of skeletal muscle. Cell Rep. (2019) 27:2029–2035.e5. doi: 10.1016/j.celrep.2019.04.074 31091443 PMC7034941

[77] Mázala DAG Novak JS Hogarth MW Nearing M Adusumalli P Tully CB . TGF-β–driven muscle degeneration and failed regeneration underlie disease onset in a DMD mouse model. JCI Insight. (2020) 5:e135703. doi: 10.1172/jci.insight.135703 32213706 PMC7213798

[78] Defour A Medikayala S Van der Meulen JH Hogarth MW Holdreith N Malatras A . Annexin A2 links poor myofiber repair with inflammation and adipogenic replacement of the injured muscle. Hum Mol Genet. (2017) 26:1979–91. doi: 10.1093/hmg/ddx065 28334824 PMC6075559

[79] Hogarth MW Defour A Lazarski C Gallardo E Diaz Manera J Partridge TA . Fibroadipogenic progenitors are responsible for muscle loss in limb girdle muscular dystrophy 2B. Nat Commun. (2019) 10:2430. doi: 10.1038/s41467-019-10438-z 31160583 PMC6547715

[80] Lemos DR Paylor B Chang C Sampaio A Underhill TM Rossi FMV . Functionally convergent white adipogenic progenitors of different lineages participate in a diffused system supporting tissue regeneration. Stem Cells. (2012) 30:1152–62. doi: 10.1002/stem.1082 22415977

[81] Deng B Wehling-Henricks M Villalta SA Wang Y Tidball JG . Interleukin-10 triggers changes in macrophage phenotype that promote muscle growth and regeneration. J Immunol. (2012) 189:3669–80. doi: 10.4049/jimmunol.1103180 22933625 PMC3448810

[82] Mozzetta C Consalvi S Saccone V Tierney M Diamantini A Mitchell KJ . Fibroadipogenic progenitors mediate the ability of HDAC inhibitors to promote regeneration in dystrophic muscles of young, but not old Mdx mice. EMBO Mol Med. (2013) 5:626–39. doi: 10.1002/emmm.201202096 23505062 PMC3628105

[83] Lukjanenko L Karaz S Stuelsatz P Gurriaran-Rodriguez U Michaud J Dammone G . Aging disrupts muscle stem cell function by impairing matricellular WISP1 secretion from fibro-adipogenic progenitors. Cell Stem Cell. (2019) 24:433–446.e7. doi: 10.1016/j.stem.2018.12.014 30686765 PMC6408230

[84] Mázala DAG Hindupur R Moon YJ Shaikh F Gamu IH Alladi D . Altered muscle niche contributes to myogenic deficit in the D2-mdx model of severe DMD. Cell Death Discov. (2023) 9:224. doi: 10.1038/s41420-023-01503-0 37402716 PMC10319851

[85] Moon YJ Hindupur R Gamu IH McCormack NM Shaikh F Novak JS . A combinatorial oligonucleotide therapy to improve dystrophin restoration and dystrophin-deficient muscle health. Mol Ther Nucleic Acids. (2025) 36:102665. doi: 10.1016/j.omtn.2025.102665 40896584 PMC12398789

[86] Kanugovi A Aguiari P Choi R Kim S Wu D De Morree A . Targeting C1q signaling in fibro-adipogenic progenitors prevents regenerative fibrosis of aged muscle. Proc Natl Acad Sci USA. (2026) 123:e2423340122. doi: 10.1073/pnas.2423340122 41505520 PMC12799171

[87] Spiller KL Anfang R Spiller KJ Ng J Nakazawa KR Daulton JW . The role of macrophage phenotype in vascularization of tissue engineering scaffolds. Biomaterials. (2014) 35:4477–88. doi: 10.1016/j.biomaterials.2014.02.012 24589361 PMC4000280

[88] Latroche C Weiss-Gayet M Muller L Gitiaux C Leblanc P Liot S . Coupling between myogenesis and angiogenesis during skeletal muscle regeneration is stimulated by restorative macrophages. Stem Cell Rep. (2017) 9:2018–33. doi: 10.1016/j.stemcr.2017.10.027 29198825 PMC5785732

[89] Tattersall IW Du J Cong Z Cho BS Klein AM Dieck CL . *In vitro* modeling of endothelial interaction with macrophages and pericytes demonstrates Notch signaling function in the vascular microenvironment. Angiogenesis. (2016) 19:201–15. doi: 10.1007/s10456-016-9501-1 26965898 PMC5576170

[90] Wu WK Georgiadis A Copland DA Liyanage S Luhmann UFO Robbie SJ . IL-4 regulates specific Arg-1+ macrophage sFlt-1–mediated inhibition of angiogenesis. Am J Pathol. (2015) 185:2324–35. doi: 10.1016/j.ajpath.2015.04.013 26079814

[91] Krieger J Ogle M McFaline-Figueroa J Segar C Temenoff J Botchwey E . Spatially localized recruitment of anti-inflammatory monocytes by SDF-1α-releasing hydrogels enhances microvascular network remodeling. Biomaterials. (2016) 77:280–90. doi: 10.1016/j.biomaterials.2015.10.045 26613543 PMC4698334

[92] Timóteo-Ferreira F Bergamaschi M Gamberale R Barone C D’Orlando C Tascini AS . Single-cell transcriptomics highlights macrophage-driven regulation of EndMT and repair in injured muscle. Commun Biol. (2026) 9:987. doi: 10.1038/s42003-026-10194-z 42115342 PMC13381571

[93] Zordan P Rigamonti E Freudenberg K Conti V Azzoni E Rovere-Querini P . Macrophages commit postnatal endothelium-derived progenitors to angiogenesis and restrict endothelial to mesenchymal transition during muscle regeneration. Cell Death Dis. (2014) 5:e1031. doi: 10.1038/cddis.2013.558 24481445 PMC4040684

[94] Iavarone F Guardiola O Scagliola A Andolfi G Esposito F Serrano A . Cripto shapes macrophage plasticity and restricts EndMT in injured and diseased skeletal muscle. EMBO Rep. (2020) 21:e49075. doi: 10.15252/embr.201949075 32107853 PMC7132341

[95] Christov C Chr étien F Abou -Khalil R Bassez G Vallet G Authier FJ . Muscle satellite cells and endothelial cells: close neighbors and privileged partners. Mol Biol Cell. (2007) 18:1397–409. doi: 10.1091/mbc.e06-08-0693 17287398 PMC1838982

[96] Yoshida T Delafontaine P . Mechanisms of IGF-1-mediated regulation of skeletal muscle hypertrophy and atrophy. Cells. (2020) 9:1970. doi: 10.3390/cells9091970 32858949 PMC7564605

[97] Yang C Zhang L Liu L Bai L Liu Y Wang R . Advances in vascular endothelial cell-mediated regulation of satellite cell repair and regeneration. Inflammation Regener. (2025) 45:30. doi: 10.1186/s41232-025-00394-1 41057951 PMC12502518

[98] Scaffidi P Misteli T Bianchi ME . Release of chromatin protein HMGB1 by necrotic cells triggers inflammation. Nature. (2002) 418:191–5. doi: 10.1038/nature00858 12110890

[99] Venereau E Casalgrandi M Schiraldi M Antoine DJ Cattaneo A De Marchis F . Mutually exclusive redox forms of HMGB1 promote cell recruitment or proinflammatory cytokine release. J Exp Med. (2012) 209:1519–28. doi: 10.1084/jem.20120189 22869893 PMC3428943

[100] Murray PJ Allen JE Biswas SK Fisher EA Gilroy DW Goerdt S . Macrophage activation and polarization: nomenclature and experimental guidelines. Immunity. (2014) 41:14–20. doi: 10.1016/j.immuni.2014.06.008 25035950 PMC4123412

[101] Novak ML Weinheimer-Haus EM Koh TJ . Macrophage activation and skeletal muscle healing following traumatic injury. J Pathol. (2014) 232:344–55. doi: 10.1002/path.4301 24255005 PMC4019602

[102] Wang X Zhao W Ransohoff RM Zhou L . Infiltrating macrophages are broadly activated at the early stage to support acute skeletal muscle injury repair. J Neuroimmunology. (2018) 317:55–66. doi: 10.1016/j.jneuroim.2018.01.004 29325905 PMC5835410

[103] Jaitin DA Adlung L Thaiss CA Weiner A Li B Descamps H . Lipid-associated macrophages control metabolic homeostasis in a Trem2-dependent manner. Cell. (2019) 178:686–698.e14. doi: 10.1016/j.cell.2019.05.054 31257031 PMC7068689

[104] Silvin A Uderhardt S Piot C Da Mesquita S Yang K Geirsdottir L . Dual ontogeny of disease-associated microglia and disease inflammatory macrophages in aging and neurodegeneration. Immunity. (2022) 55:1448–1465.e6. doi: 10.1016/j.immuni.2022.07.004 35931085

[105] Tang W Wang X Han B Jiang SH Cao H . Tumor-associated macrophages in cancer: from mechanisms to application. Mol BioMed. (2025) 6:145. doi: 10.1186/s43556-025-00396-y 41417432 PMC12717352

[106] Fabre T Barron AMS Christensen SM Asano S Bound K Lech MP . Identification of a broadly fibrogenic macrophage subset induced by type 3 inflammation. Sci Immunol. (2023) 8:eadd8945. doi: 10.1126/sciimmunol.add8945 37027478

[107] Zhang K Jagannath C . Crosstalk between metabolism and epigenetics during macrophage polarization. Epigenet Chromatin. (2025) 18:16. doi: 10.1186/s13072-025-00575-9 40156046 PMC11954343

[108] Liu Y Xu R Gu H Zhang E Qu J Cao W . Metabolic reprogramming in macrophage responses. biomark Res. (2021) 9:1. doi: 10.1186/s40364-020-00251-y 33407885 PMC7786975

[109] Van den Bossche J Baardman J de Winther MPJ . Metabolic characterization of polarized M1 and M2 bone marrow-derived macrophages using real-time extracellular flux analysis. J Vis Exp. (2015), 53424. doi: 10.3791/53424 26649578 PMC4692751

[110] Freemerman AJ Johnson AR Sacks GN Milner JJ Kirk EL Troester MA . Metabolic reprogramming of macrophages: glucose transporter 1 (GLUT1)-mediated glucose metabolism drives a proinflammatory phenotype. J Biol Chem. (2014) 289:7884–96. doi: 10.1074/jbc.M113.522037 24492615 PMC3953299

[111] Shirai T Nazarewicz RR Wallis BB Yanes RE Watanabe R Hilhorst M . The glycolytic enzyme PKM2 bridges metabolic and inflammatory dysfunction in coronary artery disease. J Exp Med. (2016) 213:337–54. doi: 10.1084/jem.20150900 26926996 PMC4813677

[112] Wang T Liu H Lian G Zhang SY Wang X Jiang C . HIF1α-induced glycolysis metabolism is essential to the activation of inflammatory macrophages. Mediators Inflammation. (2017) 2017:9029327. doi: 10.1155/2017/9029327 29386753 PMC5745720

[113] Jha AK Huang SCC Sergushichev A Lampropoulou V Ivanova Y Loginicheva E . Network integration of parallel metabolic and transcriptional data reveals metabolic modules that regulate macrophage polarization. Immunity. (2015) 42:419–30. doi: 10.1016/j.immuni.2015.02.005 25786174

[114] Lampropoulou V Sergushichev A Bambouskova M Nair S Vincent EE Loginicheva E . Itaconate links inhibition of succinate dehydrogenase with macrophage metabolic remodeling and regulation of inflammation. Cell Metab. (2016) 24:158–66. doi: 10.1016/j.cmet.2016.06.004 27374498 PMC5108454

[115] Tannahill G Curtis A Adamik J Palsson-McDermott E McGettrick A Goel G . Succinate is a danger signal that induces IL-1β via HIF-1α. Nature. (2013) 496:238–42. doi: 10.1038/nature11986 23535595 PMC4031686

[116] Littlewood-Evans A Sarret S Apfel V Loesle P Dawson J Zhang J . GPR91 senses extracellular succinate released from inflammatory macrophages and exacerbates rheumatoid arthritis. J Exp Med. (2016) 213:1655–62. doi: 10.1084/jem.20160061 27481132 PMC4995082

[117] Mills EL Kelly B Logan A Costa ASH Varma M Bryant CE . Succinate dehydrogenase supports metabolic repurposing of mitochondria to drive inflammatory macrophages. Cell. (2016) 167:457–470.e13. doi: 10.1016/j.cell.2016.08.064 27667687 PMC5863951

[118] Xiao M Yang H Xu W Ma S Lin H Zhu H . Inhibition of α-KG-dependent histone and DNA demethylases by fumarate and succinate that are accumulated in mutations of FH and SDH tumor suppressors. Genes Dev. (2012) 26:1326–38. doi: 10.1101/gad.191056.112 22677546 PMC3387660

[119] Barski A Cuddapah S Cui K Roh TY Schones DE Wang Z . High-resolution profiling of histone methylations in the human genome. Cell. (2007) 129:823–37. doi: 10.1016/j.cell.2007.05.009 17512414

[120] Guenther MG Levine SS Boyer LA Jaenisch R Young RA . A chromatin landmark and transcription initiation at most promoters in human cells. Cell. (2007) 130:77–88. doi: 10.1016/j.cell.2007.05.042 17632057 PMC3200295

[121] Santos-Rosa H Schneider R Bannister AJ Sherriff J Bernstein BE Emre NCT . Active genes are tri-methylated at K4 of histone H3. Nature. (2002) 419:407–11. doi: 10.1038/nature01080 12353038

[122] Arts RJW Novakovic B Ter Horst R Carvalho A Bekkering S Lachmandas E . Glutaminolysis and fumarate accumulation integrate immunometabolic and epigenetic programs in trained immunity. Cell Metab. (2016) 24:807–19. doi: 10.1016/j.cmet.2016.10.008 27866838 PMC5742541

[123] Cheng SC Quintin J Cramer RA Shepardson KM Saeed S Kumar V . mTOR/HIF1α-mediated aerobic glycolysis as metabolic basis for trained immunity. Science. (2014) 345:1250684. doi: 10.1126/science.1250684 25258083 PMC4226238

[124] Byles V Covarrubias AJ Ben-Sahra I Lamming DW Sabatini DM Manning BD . The TSC-mTOR pathway regulates macrophage polarization. Nat Commun. (2013) 4:2834. doi: 10.1038/ncomms3834 24280772 PMC3876736

[125] Haloul M Oliveira ERA Kader M Wells JZ Tominello TR El Andaloussi A . mTORC1-mediated polarization of M1 macrophages and their accumulation in the liver correlate with immunopathology in fatal ehrlichiosis. Sci Rep. (2019) 9:14050. doi: 10.1038/s41598-019-50320-y 31575880 PMC6773708

[126] Shen X Sun C Cheng Y Ma D Sun Y Lin Y . cGAS mediates inflammation by polarizing macrophages to M1 phenotype via the mTORC1 pathway. J Immunol. (2023) 210:1098–107. doi: 10.4049/jimmunol.2200351 36881861

[127] Yang K Xu C Zhang Y He S Li D . Sestrin2 suppresses classically activated macrophages-mediated inflammatory response in myocardial infarction through inhibition of mTORC1 signaling. Front Immunol. (2017) 8:728. doi: 10.3389/fimmu.2017.00728 28713369 PMC5491632

[128] Wang F Zhang S Vuckovic I Jeon R Lerman A Folmes CD . Glycolytic stimulation is not a requirement for M2 macrophage differentiation. Cell Metab. (2018) 28:463–475.e4. doi: 10.1016/j.cmet.2018.08.012 30184486 PMC6449248

[129] Huang SCC Everts B Ivanova Y O’Sullivan D Nascimento M Smith AM . Cell-intrinsic lysosomal lipolysis is essential for alternative activation of macrophages. Nat Immunol. (2014) 15:846–55. doi: 10.1038/ni.2956 25086775 PMC4139419

[130] Namgaladze D Brüne B . Fatty acid oxidation is dispensable for human macrophage IL-4-induced polarization. Biochim Biophys Acta. (2014) 1841:1329–35. doi: 10.1016/j.bbalip.2014.06.007 24960101

[131] Nomura M Liu J Rovira II Gonzalez-Hurtado E Lee J Wolfgang MJ . Fatty acid oxidation in macrophage polarization. Nat Immunol. (2016) 17:216–7. doi: 10.1038/ni.3366 26882249 PMC6033271

[132] Viola A Munari F Sánchez-Rodríguez R Scolaro T Castegna A . The metabolic signature of macrophage responses. Front Immunol. (2019) 10:1462. doi: 10.3389/fimmu.2019.01462 31333642 PMC6618143

[133] Zhou W Hu G He J Wang T Zuo Y Cao Y . SENP1-Sirt3 signaling promotes α-ketoglutarate production during M2 macrophage polarization. Cell Rep. (2022) 39. doi: 10.1016/j.celrep.2022.110660 35417703

[134] Liu PS Wang H Li X Chao T Teav T Christen S . α-ketoglutarate orchestrates macrophage activation through metabolic and epigenetic reprogramming. Nat Immunol. (2017) 18:985–94. doi: 10.1038/ni.3796 28714978

[135] Chen J Xu X Li Y Li F Zhang J Xu Q . Kdm6a suppresses the alternative activation of macrophages and impairs energy expenditure in obesity. Cell Death Differ. (2021) 28:1688–704. doi: 10.1038/s41418-020-00694-8 33303977 PMC8167088

[136] Ishii M Wen H Corsa CAS Liu T Coelho AL Allen RM . Epigenetic regulation of the alternatively activated macrophage phenotype. Blood. (2009) 114:3244–54. doi: 10.1182/blood-2009-04-217620 19567879 PMC2759649

[137] Kouzarides T . Chromatin modifications and their function. Cell. (2007) 128:693–705. doi: 10.1016/j.cell.2007.02.005 17320507

[138] Li X Zhang Q Shi Q Liu Y Zhao K Shen Q . Demethylase Kdm6a epigenetically promotes IL-6 and IFN-β production in macrophages. J Autoimmun. (2017) 80:85–94. doi: 10.1016/j.jaut.2017.02.007 28284523

[139] Covarrubias AJ Kale A Perrone R Lopez-Dominguez JA Pisco AO Kasler HG . Senescent cells promote tissue NAD+ decline during ageing via the activation of CD38+ macrophages. Nat Metab. (2020) 2:1265–83. doi: 10.1038/s42255-020-00305-3 33199924 PMC7908681

[140] Xu CQ Li J Liang ZQ Zhong YL Zhang ZH Hu XQ . Sirtuins in macrophage immune metabolism: a novel target for cardiovascular disorders. Int J Biol Macromol. (2024) 256:128270. doi: 10.1016/j.ijbiomac.2023.128270 38000586

[141] Feldman JL Dittenhafer-Reed KE Denu JM . Sirtuin catalysis and regulation. J Biol Chem. (2012) 287:42419–27. doi: 10.1074/jbc.R112.378877 23086947 PMC3522242

[142] Park SY Lee SW Lee SY Hong KW Bae SS Kim K . SIRT1/adenosine monophosphate-activated protein kinase α signaling enhances macrophage polarization to an anti-inflammatory phenotype in rheumatoid arthritis. Front Immunol. (2017) 8:1135. doi: 10.3389/fimmu.2017.01135 28966618 PMC5605563

[143] McArthur S Juban G Gobbetti T Desgeorges T Theret M Gondin J . Annexin A1 drives macrophage skewing to accelerate muscle regeneration through AMPK activation. J Clin Invest. (2020) 130:1156–67. doi: 10.1172/JCI124635 32015229 PMC7269594

[144] Ryall JG Dell’Orso S Derfoul A Juan A Zare H Feng X . The NAD(+)-dependent SIRT1 deacetylase translates a metabolic switch into regulatory epigenetics in skeletal muscle stem cells. Cell Stem Cell. (2015) 16:171–83. doi: 10.1016/j.stem.2014.12.004 25600643 PMC4320668

[145] Kauppinen A Suuronen T Ojala J Kaarniranta K Salminen A . Antagonistic crosstalk between NF-κB and SIRT1 in the regulation of inflammation and metabolic disorders. Cell Signalling. (2013) 25:1939–48. doi: 10.1016/j.cellsig.2013.06.007 23770291

[146] He S Jiang X Yang J Wu Y Shi J Wu X . Nicotinamide mononucleotide alleviates endotoxin-induced acute lung injury by modulating macrophage polarization via the SIRT1/NF-κB pathway. Pharm Biol. (2024) 62:22–32. doi: 10.1080/13880209.2023.2292256 38100537 PMC10732210

[147] Xiang A Chang A Zhou J Jin J Zhang X Wang Q . Macrophage metabolism in inflammatory heart disease: new insights and therapeutic implications. Front Med. (2025) 12. doi: 10.3389/fmed.2025.1664538 41133142 PMC12540094

[148] Tsai ML Tsai YG Lin YC Hsu YL Chen YT Tsai MK . IL-25 induced ROS-mediated M2 macrophage polarization via AMPK-associated mitophagy. Int J Mol Sci. (2021) 23:3. doi: 10.3390/ijms23010003 35008429 PMC8744791

[149] Feige JN Lagouge M Canto C Strehle A Houten SM Milne JC . Specific SIRT1 activation mimics low energy levels and protects against diet-induced metabolic disorders by enhancing fat oxidation. Cell Metab. (2008) 8:347–58. doi: 10.1016/j.cmet.2008.08.017 19046567

[150] Nin V Escande C Chini CC Giri S Camacho-Pereira J Matalonga J . Role of deleted in breast cancer 1 (DBC1) protein in SIRT1 deacetylase activation induced by protein kinase A and AMP-activated protein kinase *. J Biol Chem. (2012) 287:23489–501. doi: 10.1074/jbc.M112.365874 22553202 PMC3390625

[151] Cantó C Gerhart-Hines Z Feige JN Lagouge M Noriega L Milne JC . AMPK regulates energy expenditure by modulating NAD+ metabolism and SIRT1 activity. Nature. (2009) 458:1056–60. doi: 10.1038/nature07813 19262508 PMC3616311

[152] Lim JE Chung E Son Y . A neuropeptide, substance-P, directly induces tissue-repairing M2 like macrophages by activating the PI3K/Akt/mTOR pathway even in the presence of IFNγ. Sci Rep. (2017) 7:9417. doi: 10.1038/s41598-017-09639-7 28842601 PMC5573373

[153] Cai Z Li W Hager S Wilson JL Afjehi-Sadat L Heiss EH . Targeting PHGDH reverses the immunosuppressive phenotype of tumor-associated macrophages through α-ketoglutarate and mTORC1 signaling. Cell Mol Immunol. (2024) 21:448–65. doi: 10.1038/s41423-024-01134-0 38409249 PMC11061172

[154] Nagaraju K Rawat R Veszelovszky E Thapliyal R Kesari A Sparks S . Dysferlin deficiency enhances monocyte phagocytosis. Am J Pathol. (2008) 172:774–85. doi: 10.2353/ajpath.2008.070327 18276788 PMC2258254

[155] Dadgar S Wang Z Johnston H Kesari A Nagaraju K Chen YW . Asynchronous remodeling is a driver of failed regeneration in Duchenne muscular dystrophy. J Cell Biol. (2014) 207:139–58. doi: 10.1083/jcb.201402079 25313409 PMC4195829

[156] Angelini C . LGMD. Identification, description and classification. Acta Myol. (2020) 39:207–17. doi: 10.36185/2532-1900-024 33458576 PMC7783424

[157] Chen YW Bittel AJ Bittel DC Moon YJ McCormack NM Jaiswal JK . Muscular dystrophies. Adv Exp Med Biol. (2025) 1478:245–84. doi: 10.1007/978-3-031-88361-3_11 40879943

[158] Chen YW Nagaraju K Bakay M McIntyre O Rawat R Shi R . Early onset of inflammation and later involvement of TGFβ in Duchenne muscular dystrophy. Neurology. (2005) 65:826–34. doi: 10.1212/01.wnl.0000173836.09176.c4 16093456

[159] De Paepe B De Bleecker JL . Cytokines and chemokines as regulators of skeletal muscle inflammation: Presenting the case of Duchenne muscular dystrophy. Mediators Inflammation. (2013) 2013:540370. doi: 10.1155/2013/540370 24302815 PMC3835490

[160] Mercuri E Bönnemann CG Muntoni F . Muscular dystrophies. Lancet. (2019) 394:2025–38. doi: 10.1016/S0140-6736(19)32910-1 31789220

[161] Wehling-Henricks M Jordan MC Gotoh T Grody WW Roos KP Tidball JG . Arginine metabolism by macrophages promotes cardiac and muscle fibrosis in mdx muscular dystrophy. PloS One. (2010) 5:e10763. doi: 10.1371/journal.pone.0010763 20505827 PMC2874011

[162] Stec MJ Su Q Adler C Zhang L Golann DR Khan NP . A cellular and molecular spatial atlas of dystrophic muscle. Proc Natl Acad Sci USA. (2023) 120:e2221249120. doi: 10.1073/pnas.2221249120 37410813 PMC10629561

[163] Scripture-Adams DD Chesmore KN Barthélémy F Wang RT Nieves-Rodriguez S Wang DW . Single nuclei transcriptomics of muscle reveals intra-muscular cell dynamics linked to dystrophin loss and rescue. Commun Biol. (2022) 5:989. doi: 10.1038/s42003-022-03938-0 36123393 PMC9485160

[164] Wu Z Yu S Hu Y Hu X Yang L Jiang Y . TREM2 facilitates myelin debris clearance but exacerbates chronic inflammation and fibrosis after spinal cord injury. CNS Neurosci Ther. (2026) 32:e70777. doi: 10.1002/cns.70777 41660680 PMC12884443

[165] King EM Zhao Y Moore CM Steinhart B Anderson KC Vestal B . Gpnmb and Spp1 mark a conserved macrophage injury response masking fibrosis-specific programming in the lung. JCI Insight. (2024) 9:e182700. doi: 10.1172/jci.insight.182700 39509324 PMC11665561

[166] Novak R Dabelic S Dumic J . Galectin-1 and galectin-3 expression profiles in classically and alternatively activated human macrophages. Biochim Biophys Acta (BBA) - Gen Subj. (2012) 1820:1383–90. doi: 10.1016/j.bbagen.2011.11.014 22155450

[167] Ripoll VM Irvine KM Ravasi T Sweet MJ Hume DA . Gpnmb is induced in macrophages by IFN-γ and lipopolysaccharide and acts as a feedback regulator of proinflammatory responses1. J Immunol. (2007) 178:6557–66. doi: 10.4049/jimmunol.178.10.6557 17475886

[168] Cerri DG Rodrigues LC Alves VM MaChado J Bastos VAF Carmo Kettelhut I . Endogenous galectin-3 is required for skeletal muscle repair. Glycobiology. (2021) 31:1295–307. doi: 10.1093/glycob/cwab071 34224566

[169] Palma A . The landscape of SPP1 + macrophages across tissues and diseases: A comprehensive review. Immunology. (2025) 176:179–96. doi: 10.1111/imm.13952 40395129 PMC12401812

[170] Al-Dalahmah O Lam M McInvale JJ Qu W Nguyen T Mun JY . Osteopontin drives neuroinflammation and cell loss in MAPT-N279K frontotemporal dementia patient neurons. Cell Stem Cell. (2024) 31:676–693.e10. doi: 10.1016/j.stem.2024.03.013 38626772 PMC11373574

[171] Huang HY Chen YZ Zhao C Zheng XN Yu K Yue JX . Alternations in inflammatory macrophage niche drive phenotypic and functional plasticity of Kupffer cells. Nat Commun. (2024) 15:9337. doi: 10.1038/s41467-024-53659-7 39472435 PMC11522483

[172] Ramachandran P Dobie R Wilson-Kanamori JR Dora EF Henderson BEP Luu NT . Resolving the fibrotic niche of human liver cirrhosis at single-cell level. Nature. (2019) 575:512–8. doi: 10.1038/s41586-019-1631-3 31597160 PMC6876711

[173] Morse C Tabib T Sembrat J Buschur KL Bittar HT Valenzi E . Proliferating SPP1/MERTK-expressing macrophages in idiopathic pulmonary fibrosis. Eur Respir J. (2019) 54. doi: 10.1183/13993003.02441-2018 31221805 PMC8025672

[174] Vannan A Lyu R Williams AL Negretti NM Mee ED Hirsh J . Spatial transcriptomics identifies molecular niche dysregulation associated with distal lung remodeling in pulmonary fibrosis. Nat Genet. (2025) 57:647–58. doi: 10.1038/s41588-025-02080-x 39901013 PMC11906353

[175] Liu Y Zhang Q Xing B Luo N Gao R Yu K . Immune phenotypic linkage between colorectal cancer and liver metastasis. Cancer Cell. (2022) 40:424–437.e5. doi: 10.1016/j.ccell.2022.02.013 35303421

[176] Sousa NS Bica M Brás MF Sousa AC Antunes IB Encarnação IA . The immune landscape of murine skeletal muscle regeneration and aging. Cell Rep. (2024) 43:114975. doi: 10.1016/j.celrep.2024.114975 39541212

[177] Capote J Kramerova I Martinez L Vetrone S Barton ER Sweeney HL . Osteopontin ablation ameliorates muscular dystrophy by shifting macrophages to a pro-regenerative phenotype. J Cell Biol. (2016) 213:275–88. doi: 10.1083/jcb.201510086 27091452 PMC5084275

[178] Behmoaras J Mulder K Ginhoux F Petretto E . The spatial and temporal activation of macrophages during fibrosis. Nat Rev Immunol. (2025) 25:816–30. doi: 10.1038/s41577-025-01186-x 40467841

[179] Hoeft K Schaefer GJL Kim H Schumacher D Bleckwehl T Long Q . Platelet-instructed SPP1+ macrophages drive myofibroblast activation in fibrosis in a CXCL4-dependent manner. Cell Rep. (2023) 42:112131. doi: 10.1016/j.celrep.2023.112131 36807143 PMC9992450

[180] Kramerova I Kumagai-Cresse C Ermolova N Mokhonova E Marinov M Capote J . Spp1 (osteopontin) promotes TGFβ processing in fibroblasts of dystrophin-deficient muscles through matrix metalloproteinases. Hum Mol Genet. (2019) 28:3431–42. doi: 10.1093/hmg/ddz181 31411676 PMC7345878

[181] Wang X Wang Y Yang L Zhang Y Yang L . TREM2+ macrophages: a key role in disease development. Front Immunol. (2025) 16. doi: 10.3389/fimmu.2025.1550893 40242752 PMC12000036

[182] Luo Q Deng D Li Y Shi H Zhao J Qian Q . TREM2 insufficiency protects against pulmonary fibrosis by inhibiting M2 macrophage polarization. Int Immunopharmacol. (2023) 118:110070. doi: 10.1016/j.intimp.2023.110070 37003186

[183] Zhang J Liu Y Zheng Y Luo Y Du Y Zhao Y . TREM-2-p38 MAPK signaling regulates neuroinflammation during chronic cerebral hypoperfusion combined with diabetes mellitus. J Neuroinflamm. (2020) 17:2. doi: 10.1186/s12974-019-1688-9 31900229 PMC6942413

[184] Patterson MT Firulyova MM Xu Y Hillman H Bishop C Zhu A . Trem2 promotes foamy macrophage lipid uptake and survival in atherosclerosis. Nat Cardiovasc Res. (2023) 2:1015. doi: 10.1038/s44161-023-00354-3 38646596 PMC11031198

[185] Zhou L Qiu X Meng Z Liu T Chen Z Zhang P . Hepatic danger signaling triggers TREM2+ macrophage induction and drives steatohepatitis via MS4A7-dependent inflammasome activation. Sci Transl Med. (2024) 16:eadk1866. doi: 10.1126/scitranslmed.adk1866 38478630 PMC12287971

[186] Park M Yi JW Kim EM Yoon IJ Lee EH Lee HY . Triggering receptor expressed on myeloid cells 2 (TREM2) promotes adipogenesis and diet-induced obesity. Diabetes. (2015) 64:117–27. doi: 10.2337/db13-1869 25114293

[187] Blasetti Fantauzzi C Iacobini C Menini S Vitale M Sorice GP Mezza T . Galectin-3 gene deletion results in defective adipose tissue maturation and impaired insulin sensitivity and glucose homeostasis. Sci Rep. (2020) 10:20070. doi: 10.1038/s41598-020-76952-z 33208796 PMC7675972

[188] Farahat PK Kumagai-Cresse C Aragón RL Ma F Amakor JK Espinoza A . Macrophage-derived Spp1 promotes intramuscular fat in dystrophic muscle. JCI Insight. (2025) 10:e181946. doi: 10.1172/jci.insight.181946 40626359 PMC12288893

[189] Lei L Wang X Zhao J . SPP1 expression after spinal cord compression injury and its effects on glial cell activation. Neurochem Int. (2025) 190:106063. doi: 10.1016/j.neuint.2025.106063 41015119

[190] Moore SM Holt VV Malpass LR Hines IN Wheeler MD . Fatty acid-binding protein 5 limits the anti-inflammatory response in murine macrophages. Mol Immunol. (2015) 67:265–75. doi: 10.1016/j.molimm.2015.06.001 26105806 PMC4565774

[191] Yin P Chen M Rao M Lin Y Zhang M Xu R . Deciphering immune landscape remodeling unravels the underlying mechanism for synchronized muscle and bone aging. Adv Sci (Weinh). (2023) 11:2304084. doi: 10.1002/advs.202304084 38088531 PMC10837389

[192] Hou Y Wei D Zhang Z Guo H Li S Zhang J . FABP5 controls macrophage alternative activation and allergic asthma by selectively programming long-chain unsaturated fatty acid metabolism. Cell Rep. (2022) 41:111668. doi: 10.1016/j.celrep.2022.111668 36384126

[193] Biggar WD Skalsky A McDonald CM . Comparing deflazacort and prednisone in Duchenne muscular dystrophy. J Neuromuscul Dis. (2022) 9:463–76. doi: 10.3233/JND-210776 35723111 PMC9398085

[194] McDonald CM Gordish-Dressman H Henricson EK Duong T Joyce NC Jhawar S . Longitudinal pulmonary function testing outcome measures in Duchenne muscular dystrophy: Long-term natural history with and without glucocorticoids. Neuromuscul Disord. (2018) 28:897–909. doi: 10.1016/j.nmd.2018.07.004 30336970

[195] McDonald CM Henricson EK Abresch RT Duong T Joyce NC Hu F . Long-term effects of glucocorticoids on function, quality of life, and survival in patients with Duchenne muscular dystrophy: a prospective cohort study. Lancet. (2018) 391:451–61. doi: 10.1016/S0140-6736(17)32160-8 29174484

[196] Sali A Guerron AD Gordish-Dressman H Spurney CF Iantorno M Hoffman EP . Glucocorticoid-treated mice are an inappropriate positive control for long-term preclinical studies in the mdx mouse. PloS One. (2012) 7:e34204. doi: 10.1371/journal.pone.0034204 22509280 PMC3317932

[197] Quattrocelli M Zelikovich AS Salamone IM Fischer JA McNally EM . Mechanisms and clinical applications of glucocorticoid steroids in muscular dystrophy. J Neuromuscul Dis. (2021) 8:39–52. doi: 10.3233/JND-200556 33104035 PMC7902991

[198] Permpoon U Moon J Kim CY Nam T . Glucocorticoid-mediated skeletal muscle atrophy: Molecular mechanisms and potential therapeutic targets. Int J Mol Sci. (2025) 26:7616. doi: 10.3390/ijms26157616 40806744 PMC12347798

[199] Xie Y Tolmeijer S Oskam JM Tonkens T Meijer AH Schaaf MJM . Glucocorticoids inhibit macrophage differentiation towards a pro-inflammatory phenotype upon wounding without affecting their migration. Dis Model Mech. (2019) 12:dmm037887. doi: 10.1242/dmm.037887 31072958 PMC6550045

[200] Perretti M Montero-Melendez T . Resolution pharmacology: State-of-the-art and therapeutic landscape. Pharmacol Rev. (2025) 77:100097. doi: 10.1016/j.pharmr.2025.100097 41205263 PMC12799450

[201] Krepel SA Wang JM . Chemotactic ligands that activate G-protein-coupled formylpeptide receptors. Int J Mol Sci. (2019) 20:3426. doi: 10.3390/ijms20143426 31336833 PMC6678346

[202] Tylek K Trojan E Regulska M Lacivita E Leopoldo M Basta-Kaim A . Formyl peptide receptor 2, as an important target for ligands triggering the inflammatory response regulation: a link to brain pathology. Pharmacol Rep. (2021) 73:1004–19. doi: 10.1007/s43440-021-00271-x 34105114 PMC8413167

[203] Cooray SN Gobbetti T Montero-Melendez T McArthur S Thompson D Clark AJL . Ligand-specific conformational change of the G-protein–coupled receptor ALX/FPR2 determines proresolving functional responses. Proc Natl Acad Sci. (2013) 110:18232–7. doi: 10.1073/pnas.1308253110 24108355 PMC3831442

[204] Damazo AS Yona S D’Acquisto F Flower RJ Oliani SM Perretti M . Critical protective role for Annexin 1 gene expression in the endotoxemic murine microcirculation. Am J Pathol. (2005) 166:1607–17. doi: 10.1016/S0002-9440(10)62471-6 15920146 PMC1602430

[205] García RA Lupisella JA Ito BR Hsu MY Fernando G Carson NL . Selective FPR2 agonism promotes a proresolution macrophage phenotype and improves cardiac structure-function post myocardial infarction. JACC Basic Transl Sci. (2021) 6:676–89. doi: 10.1016/j.jacbts.2021.07.007 34466754 PMC8385569

[206] Novak JS Lischin A Uapinyoying P Hindupur R Moon YJ Bhattacharya S . Failure to resolve inflammation contributes to juvenile onset cardiac damage in a mouse model of Duchenne muscular dystrophy. Cell Death Dis. (2025) 16:505. doi: 10.1038/s41419-025-07816-5 40634289 PMC12241640

[207] Markworth JF Brown LA Lim E Floyd C Larouche J Castor-Macias JA . Resolvin D1 supports skeletal myofiber regeneration via actions on myeloid and muscle stem cells. JCI Insight. (2020) 5:e137713. doi: 10.1172/jci.insight.137713 32750044 PMC7526543

[208] Peruzzi E Heidenreich S Klaus L Boshnakovska A Amouret A Legler T . Glucocorticoids induce an opposite metabolic switch in human monocytes contingent upon their polarization. Biomolecules. (2025) 15:1422. doi: 10.3390/biom15101422 41154651 PMC12564316

[209] Diaz‐Jimenez D Oakley RH Hoffman JA Bortner CD Lih F Li J . Timing of glucocorticoid treatment dictates glucocorticoid receptor actions modulating the NLRP3‐inflammasome activation in macrophages. FASEB J. (2026) 40:e71510. doi: 10.1096/fj.202504083R 41623187 PMC12862736

[210] Stifel U Wolfschmitt EM Vogt J Wachter U Vettorazzi S Tews D . Glucocorticoids coordinate macrophage metabolism through the regulation of the tricarboxylic acid cycle. Mol Metab. (2021) 57:101424. doi: 10.1016/j.molmet.2021.101424 34954109 PMC8783148

[211] Auger JP Zimmermann M Faas M Stifel U Chambers D Krishnacoumar B . Metabolic rewiring promotes anti-inflammatory effects of glucocorticoids. Nature. (2024) 629:184–92. doi: 10.1038/s41586-024-07282-7 38600378

[212] Caratti G Desgeorges T Juban G Stifel U Fessard A Koenen M . Macrophagic AMPKα1 orchestrates regenerative inflammation induced by glucocorticoids. EMBO Rep. (2022) 24:e55363. doi: 10.15252/embr.202255363 36520372 PMC9900347

[213] Bonkowski MS Sinclair DA . Slowing ageing by design: the rise of NAD+ and sirtuin-activating compounds. Nat Rev Mol Cell Biol. (2016) 17:679–90. doi: 10.1038/nrm.2016.93 27552971 PMC5107309

[214] Jang S Kang HT Hwang ES . Nicotinamide-induced mitophagy: event mediated by high NAD+/NADH ratio and SIRT1 protein activation*. J Biol Chem. (2012) 287:19304–14. doi: 10.1074/jbc.M112.363747 22493485 PMC3365962

[215] Miyaji N Nishida K Tanaka T Araki D Kanzaki N Hoshino Y . Inhibition of knee osteoarthritis progression in mice by administering SRT2014, an activator of silent information regulator 2 ortholog 1. Cartilage. (2021) 13:1356S–66S. doi: 10.1177/1947603519900795 31989845 PMC8804762

[216] Chang N Li J Lin S Zhang J Zeng W Ma G . Emerging roles of SIRT1 activator, SRT2104, in disease treatment. Sci Rep. (2024) 14:5521. doi: 10.1038/s41598-024-55923-8 38448466 PMC10917792

[217] Giovarelli M Zecchini S Casati SR Lociuro L Gjana O Mollica L . The SIRT1 activator SRT2104 exerts exercise mimetic effects and promotes Duchenne muscular dystrophy recovery. Cell Death Dis. (2025) 16:259. doi: 10.1038/s41419-025-07595-z 40195304 PMC11977210

[218] Sebori R Kuno A Hosoda R Hayashi T Horio Y . Resveratrol decreases oxidative stress by restoring mitophagy and improves the pathophysiology of dystrophin-deficient mdx mice. Oxid Med Cell Longev. (2018) 2018:9179270. doi: 10.1155/2018/9179270 30510631 PMC6231358

[219] Scarano N Brullo C Musumeci F Millo E Bruzzone S Schenone S . Recent advances in the discovery of SIRT1/2 inhibitors via computational methods: a perspective. Pharm (Basel). (2024) 17:601. doi: 10.3390/ph17050601 38794171 PMC11123952

